# Deficient autophagy in retinal ganglion cells impairs the degradation of intracellular organelles, leading to neurodegeneration

**DOI:** 10.1038/s41419-026-08927-3

**Published:** 2026-05-30

**Authors:** Yogapriya Sundaresan, Prabhavathi Maddineni, Sarahi Rios Arguello, Balasankara Reddy Kaipa, Justin Dinh Tran, Linya Li, Bindu Kodati, Gulab S. Zode

**Affiliations:** 1 Gavin Herbert Eye Institute- Brunson Center for Translational Vision Research, Department of Ophthalmology and Visual Sciences, University of California, Irvine, CA USA; 2 Department of Ophthalmology, School of Medicine, University of Missouri, Columbia, MO USA; 3 Department of Physiology and Biophysics, University of California, Irvine, School of Medicine, Irvine, CA USA; 4 Department of Pharmacology and Neuroscience, North Texas Eye Research Institute, University of North Texas Health Science Center, Fort Worth, TX USA

**Keywords:** Cell death in the nervous system, Retina

## Abstract

Autophagy is a fundamental catabolic process that facilitates the degradation and recycling of cellular components like protein aggregates and defective organelles. However, the precise role of constitutive autophagy in regulating retinal ganglion cell (RGC) function and survival remains largely undefined. Here, we demonstrate that RGCs exhibit a robust and highly active constitutive autophagy. Furthermore, the selective autophagy knockout in RGCs induces neurodegeneration in *Atg7*^f/f^ and *Atg5*^f/f^ conditional knockout mice. Deficient autophagy, induced by AAV2-Cre in *Atg7*^*f/f*^, *Atg5*^*f/f*^ mice, or by tamoxifen treatment in *Atg7*^*f/-*^; Nestin-CreERT2^+^ mice, resulted in significant and progressive functional and structural loss of RGCs and optic nerve degeneration. Immunostaining and transmission electron microscopic analysis revealed that deficient autophagy in RGCs led to the accumulation of damaged organelles, including swollen mitochondria, distended endoplasmic reticulum, synaptic vesicles, and enlarged Golgi apparatus within the RGC soma. These pathological changes were associated with increased p62, LC3B, and incomplete autophagosomes in the RGC soma. Notably, mass spectrometry analysis identified the accumulation of proteins associated with intracellular organelles, cellular architecture, the cytoplasm, and the ribonucleoprotein complex. Our findings indicate that deficient autophagy in RGCs results in the accumulation of defective organelles within the RGC soma, ultimately contributing to neurodegeneration.

## Introduction

The autophagy-lysosome pathway is a major clearance mechanism responsible for the degradation and recycling of intracellular contents. Macroautophagy (referred to as autophagy from now on) is the primary type of autophagy among various subtypes. Autophagy facilitates the degradation of long-lived proteins and other cellular components, such as aged/defective organelles. This mechanism consists of a series of molecular events tightly regulated by a family of proteins called autophagy-related (Atg) proteins. The autophagosome, a double-membraned vesicle, plays a crucial role in the autophagic process. This intermediate structure sequesters nonessential cellular materials and degrades them via lysosomes. Autophagy is an essential quality control mechanism to maintain cellular homeostasis [1–3].

Autophagic degradation of abnormal cellular components, including long-lived proteins and defective organelles, is critical in non-dividing neurons like retinal ganglion cells (RGCs) since they lack the ability to dilute the aggregates by cell division. RGCs are projection neurons of the retina and the only cellular component connecting other retinal neurons and the brain [4–6]. They are specialized cells exhibiting a compartmentalized architecture, with their cell bodies located in the ganglion cell layer and axons running in the nerve fiber layer. These output neurons integrate and process extensive nerve impulses from their retinal counterparts and convey them to the visual centers of the brain. Owing to their heterogeneous structure, RGCs can be divided into four subcellular components: (i) the soma, (ii) the dendrites, (iii) the unmyelinated axon, and (iv) the myelinated axon. All vital organelles, proteins, and neurotransmitters are synthesized in the soma and transported to the specific region [7, 8]. Dendrites possess a minimal ability to produce macromolecules, whereas axons completely lack the synthetic ability and predominantly depend on the soma for the biogenesis of organelles and proteins. On the other hand, aged proteins and organelles are transported back to the soma from various compartments for degradation since the soma houses the robust cellular machinery for lysosomal degradation. This includes autophagy and proteolytic processes like the ubiquitin-proteasome system [7, 8]. Due to their post-mitotic nature, functional complexity, and compartmentation, RGCs are highly susceptible to accumulating misfolded protein aggregates and dysfunctional organelles under physiological and pathological conditions. Therefore, periodic removal of cellular debris is critical for maintaining neuronal homeostasis, which predominantly depends on catabolic pathways like autophagy [9].

Defective autophagy is frequently linked with several human diseases, including neurodegenerative conditions. Neurons of the central nervous system (CNS) rely predominantly on basal autophagy. Disruption of autophagy in the CNS leads to neurodegeneration and contributes to behavioral defects in mice [10, 11]. Numerous studies have investigated the role of autophagy in glaucomatous neurodegeneration, particularly in the context of elevated intraocular pressure (IOP), a major risk factor for glaucoma and optic nerve (ON) injury. These studies demonstrate that autophagy is activated in RGCs in response to IOP elevation and ON crush [12–17]. Although these models provide valuable insights into stress-induced autophagic responses, the precise role of constitutive autophagy in RGCs under normal physiological conditions remains unexplored. We hypothesize that functional autophagy is required for RGC function and survival, and the loss of autophagy in RGC results in neurodegeneration. To test this hypothesis, we conditionally knocked out autophagy in RGCs by deleting vital autophagy regulators, *Atg5* and *Atg7*. These proteins are essential in autophagosome biogenesis*. Atg5* is a critical component of the *Atg5-Atg12-Atg16* conjugation system, while *Atg7* is an E1-like ligase required for conjugating *Atg5* with *Atg12* [18]. Here, we show that RGC-specific deletion of either *Atg5* or *Atg7* leads to deficient autophagy and RGC degeneration. Notably, deficient autophagy in RGCs leads to the accumulation of defective organelles, vesicles, and protein aggregates in the soma of RGCs, contributing to neurodegeneration.

## Results

### Autophagy-related markers are prominently expressed in RGCs under physiological conditions

**Fig. 1:**
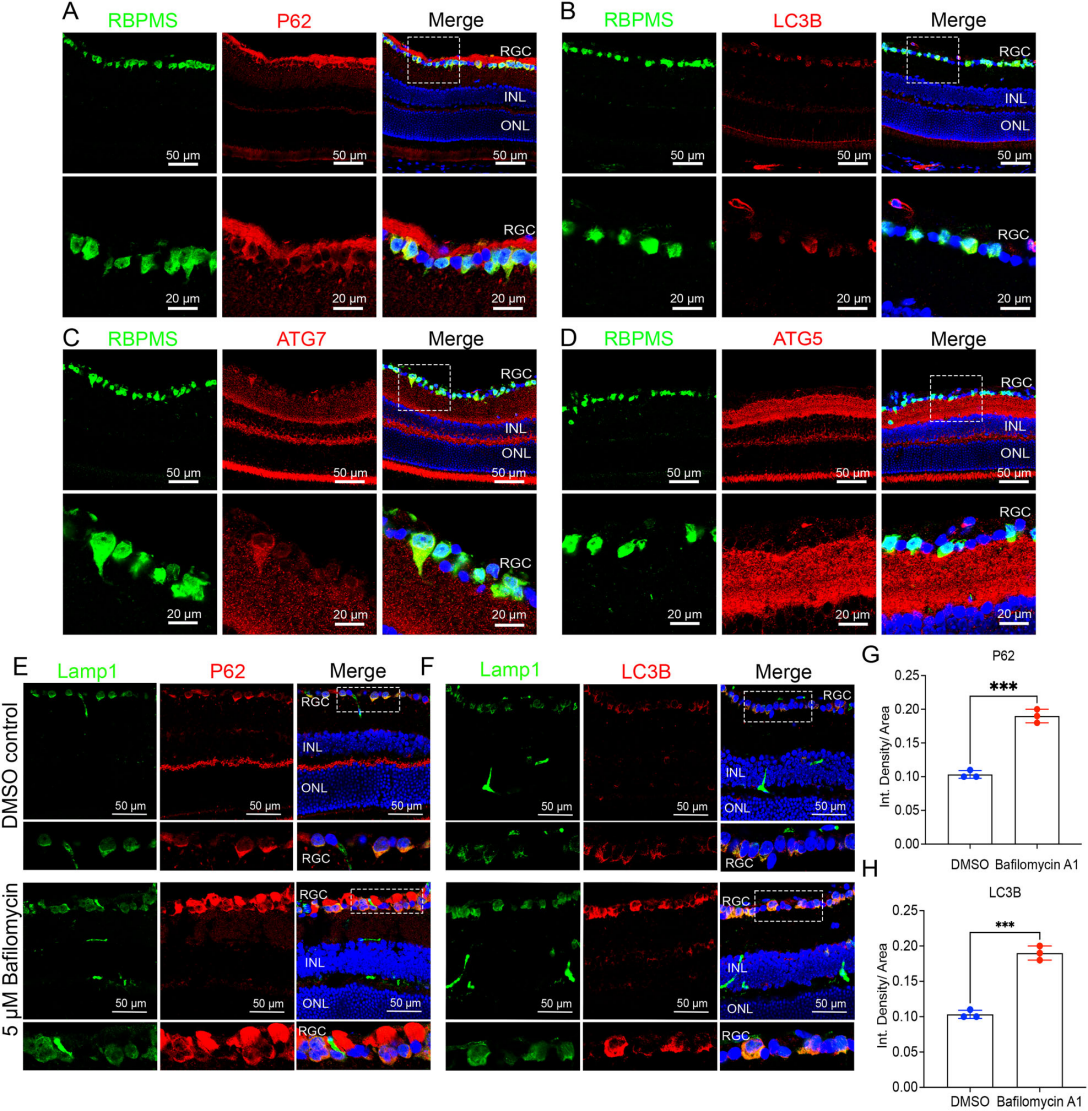
Autophagy markers in the ganglion cell layer and flux revealed by bafilomycin. Representative confocal microscopic images reveal that p62 (**A**), LC3B (**B**), ATG7 (**C**), and ATG5 (**D**) (red) are enriched in cells within the ganglion cell layer under physiological conditions. Following 2 h of in vivo bafilomycin treatment, p62 (**E**) and LC3B (**F**) (red) accumulate in the ganglion cell layer, consistent with ongoing basal autophagy revealed by lysosomal blockade. Sections are co-stained with LAMP1 (**E, F**) (green) as indicated and counterstained with DAPI (blue). Relative fluorescence intensity of p62 (**G**) and LC3B (**H**) is elevated in Bafilomycin-treated eyes compared to DMSO controls. The region outlined by the dashed box is shown at higher magnification below each panel. *n* = 3 eyes per group. Statistical test: Unpaired t-test. *** represents *p*-value ≤ 0.001. Scale bars, 50 µm; insets, 20 µm (**A**–**D**).

To define baseline distribution of autophagy machinery, we co-immunolabeled C57BL/6 J retinal sections (*n* = 3) for RBPMS and autophagy markers. p62/SQSTM1 showed a strong signal in the RGC layer and within RBPMS⁺ somata. LC3B-positive puncta were evident in RGC somata (Fig. 1A, B). ATG7, an E1-like enzyme that activates LC3/ATG8 for conjugation, and ATG5, which forms the ATG12-ATG5 complex functioning as an E3-like enzyme to promote ATG8 lipidation and autophagosome formation [19, 20], were likewise enriched in RBPMS⁺ cells (Fig. 1C, D). Together, these data indicate the prominent expression of autophagy-related proteins within RGCs in vivo, under normal physiological conditions.

### In vivo confirmation of RGC autophagic flux by bafilomycin blockade

To further confirm basal autophagy activity in RGCs, 3-month-old C57BL/6 J mice received intravitreal bafilomycin A1 (5 µM) or DMSO (control) and were analyzed 2 h later. Bafilomycin inhibits lysosomal acidification and blocks autophagosome-lysosome degradation, leading to accumulation of p62 and LC3B in the ganglion cell layer [21]. Sections were co-immunostained with LAMP1 to mark lysosomes. Confocal microscopic images and quantification (*n* = 3 eyes/group) showed increased p62 (Fig. 1E, G) and LC3B signals (Fig. 1F, H) after bafilomycin relative to vehicle, consistent with ongoing basal autophagic flux in RGCs revealed by acute lysosomal blockade.

### Selective RGC transduction by AAV2-Cre and absence of Cre toxicity in vivo

Next, we sought to knock out autophagy selectively from RGCs in *Atg7*^*f/f*^ and *Atg5*^*f/f*^ mice using AAV2-Cre. Previous studies from our lab have shown that intravitreal injection of AAV2 exhibits a robust and selective tropism to RGCs [22]. To assess AAV2 tropism for RGCs, Ai14 (Rosa-tdTomato) reporter mice were intravitreally injected with AAV2-Cre driven by CMV or Synapsin (SYN) (*n* = 3 eyes/group). Five weeks later, cryosections showed tdTomato signal concentrated in the ganglion cell layer and co-localized with the RGC marker TUJ1, confirming RGC-enriched transduction by both constructs (Fig. S1A). In addition, retinal flat mounts were stained for RGC marker, RBPMS, to quantify the percentage of RGCs expressing tdTomato. Out of 100 RBPMS^+^ RGCs, 77.00 ± 3.28 and 83.9 ± 2.44 RGCs expressed tdTomato in AAV2-Cre (CMV) and AAV2-Cre(SYN) injected eyes, respectively (Fig. S1B). While CMV drove efficient expression in RGCs, it also produced rare INL labeling ( < 0.5% of INL cells). In contrast, SYN-driven expression was confined to the RGC layer with no detectable signal in other retinal cells. Consistent results were obtained in C57BL/6 eyes injected with AAV2-eGFP (CMV or SYN) (Fig. S1C).

To assess Cre toxicity, C57BL/6 mice received AAV2-null or AAV2-Cre and were analyzed 10 weeks post-injection. Cre toxicity was assessed 10 weeks post-injection to match the primary experimental endpoint. PERG amplitudes were unchanged between groups (Fig. S2A), and quantitative immunostaining for p62 (Fig. S2B, C), LC3B (Fig. S2B, D), and COX4 (Fig. S2B, E) showed no differences, indicating that Cre expression does not impair RGC function or basal marker levels. Thus, the phenotypes in *Atg7*^*f/f*^ and *Atg5*^*f/f*^ eyes will potentially reflect loss of autophagy genes, not Cre-related effects.

### Impaired autophagy leads to functional and structural loss of RGCs and degeneration of ON axons in *Atg7*^*f*/*f*^ mice

**Fig. 2:**
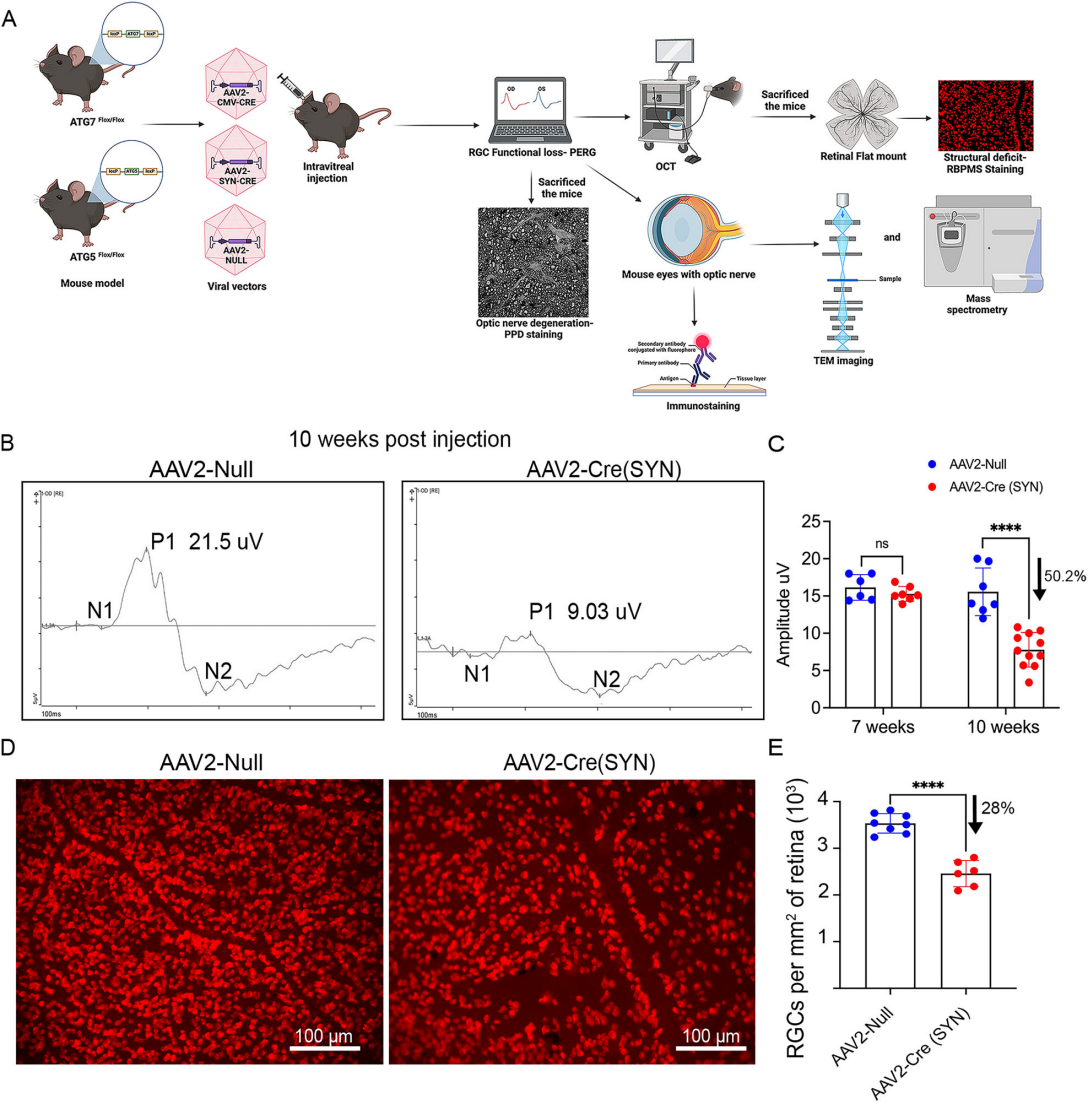
Deficient autophagy leads to functional and structural loss of RGCs in *Atg7*^*f/f*^ mice. **A** The schematic illustrates the workflow for Cre-mediated deletion of *Atg7/Atg5* in adult RGCs. For the data shown, *Atg7*^*f/f*^ mice received intravitreal AAV2-Cre under the Synapsin (SYN) promoter. **B** Representative PERG waveforms at 10 weeks post-injection from AAV2-null and AAV2-Cre(SYN) eyes; 7-week traces are not shown because there was no difference between groups. **C** Quantification of PERG P1 amplitudes shows no change at 7 weeks but a significant reduction at 10 weeks in Cre(SYN) eyes. **D** Representative RBPMS-labeled retinal whole mounts at 10 weeks. **E** Quantification of total RGC counts at 10 weeks shows a significant decrease in Cre(SYN) eyes. Statistical tests: (C) two-way ANOVA with multiple comparisons; **E** unpaired t-test. PERG: Null, *n* = 13 eyes; Cre(SYN), *n* = 18 eyes. RBPMS: Null, *n* = 8 eyes; Cre(SYN), *n* = 6 eyes. Scale bars, 100 µm. Significance: ns, not significant; ****, *p* ≤ 0.0001.

To test whether autophagy loss compromises RGC integrity, *Atg7*^*f/f*^ eyes received AAV2-Cre driven by the Synapsin promoter or AAV2-Null and were evaluated at seven and ten weeks following the workflow in Fig. 2A. Under Synapsin, RGC function was unchanged at seven weeks but showed a significant decrease (50.2%) in PERG P1 amplitude by ten weeks (Fig. 2B, C), indicating progressive functional impairment. Structural analyses at ten weeks demonstrated an overall reduction in total RGC counts on RBPMS whole mounts (28% decrease) (Fig. 2D, E) and thinning of the retinal nerve fiber layer (RNFL) on optical coherence tomography (OCT) imaging (9.9 ± 0.45 µm versus 11.06 ± 0.18 µm in controls, *p* < 0.0001) (Fig. S3F, G). To verify that these phenotypes were not promoter dependent, we repeated the experiments with AAV2-Cre driven by the CMV promoter and present those data in the Supplementary Fig. (Fig. S3). In the CMV cohort, RGC transduction was robust, and the phenotype appeared earlier. PERG revealed reduced P1 amplitude by three weeks, with a further decline by seven weeks (Fig. S3A). RBPMS immunostaining on retinal flat mounts demonstrated a 32.2% reduction in total RGC counts, and paraphenylenediamine (PPD) staining of the ON showed a 36.5% decrease in axon number, together with degenerative features (Fig. S3B–D). OCT confirmed RNFL thinning to 9.075 ± 0.16 µm compared with 11.06 ± 0.18 µm in controls (*p* < 0.0001) (Fig. S3F, G). Because the CMV promoter exhibits broader retinal tropism with rare INL labeling (Fig. S1), whereas the Synapsin promoter provides higher RGC specificity despite a later onset, we used the Synapsin cohort for subsequent experiments.

### Defective autophagic flux and mitochondrial accumulation in RGCs following *Atg7* deletion

**Fig. 3:**
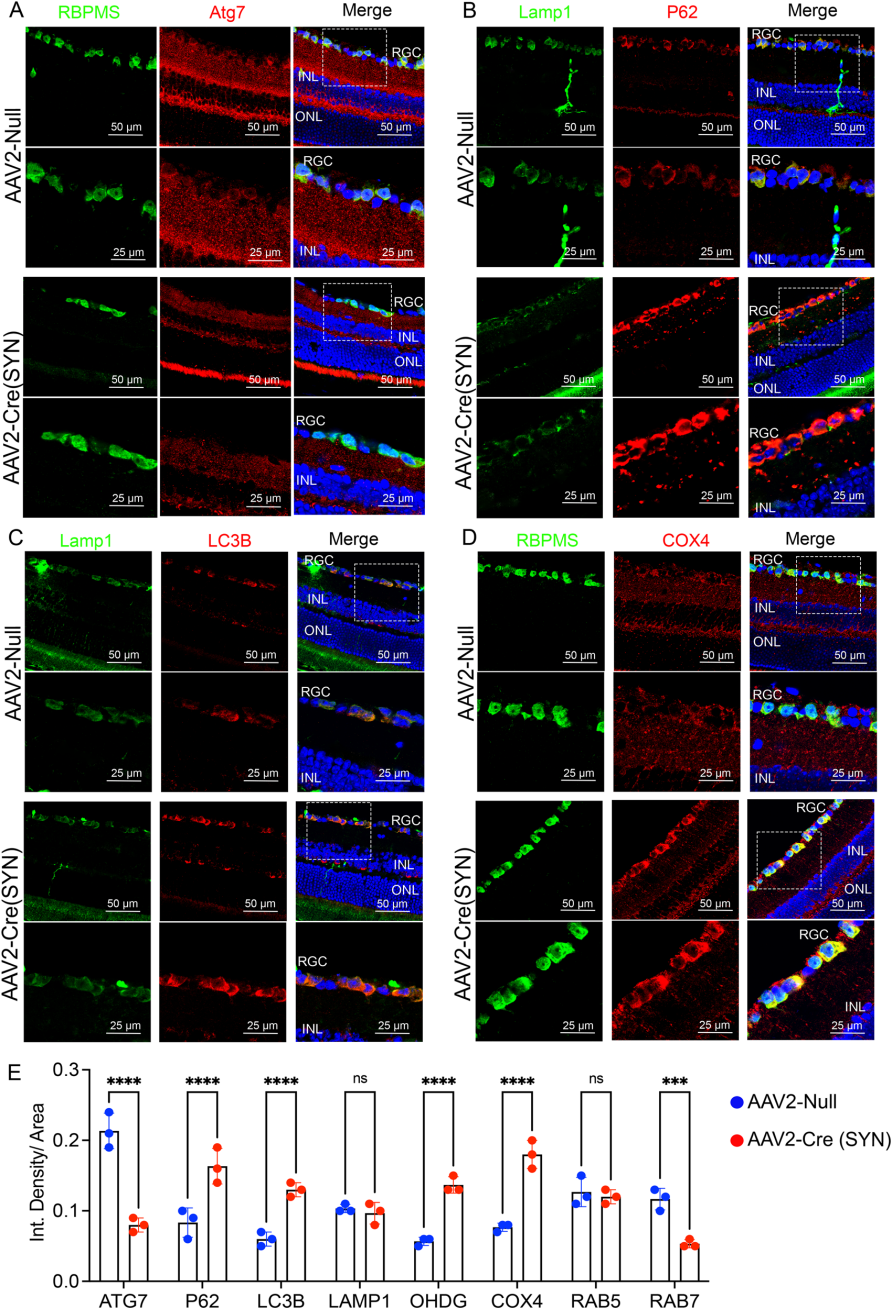
Protein levels of autophagy and mitochondrial markers in *Atg7* deficient RGCs. Representative confocal microscopic images of *Atg7*^*f/f*^ retinal sections stained for autophagy- ATG7 (**A**), p62 (**B**), LC3B (**C**) and mitochondrial-COX4 (**D**) markers (red) costained with either RGC marker, RBPMS or lysosomal marker LAMP1 (green). **E** Relative fluorescence intensity of autophagy and mitochondrial markers. The relative fluorescence intensity was significantly reduced for ATG7 in the RGC layer of Cre-injected eyes. Consistent with deficient autophagy, the relative fluorescence intensity for p62, LC3B, and COX4 was significantly higher in AAV2-Cre(SYN)-injected *Atg7*^*f/f*^ mice compared to null-injected controls. Bar-graph quantification also reveals a significant increase in 8-OHdG, indicative of elevated oxidative stress, along with preserved Rab5 and reduced Rab7 following *Atg7* deletion. Their corresponding representative images are provided in Fig. S4. The region outlined by the dashed box is shown at higher magnification below each panel. Statistical test: Two way- ANOVA with multiple comparisons. *n* = 3 eyes per group; Scale bar 50 μm, insets 25 μm. ns- not significant, *** represents *p*-value ≤ 0.001 and **** represents *p*-value ≤ 0.0001.

To confirm *Atg7* knockout and assess downstream effects, we immunostained 10-week retinal cross-sections from *Atg7*^*f/f*^ mice (AAV2-(SYN) Cre vs AAV2-null) for ATG7, p62, LC3B, COX4, 8-OHdG, Rab5, and Rab7. ATG7 immunoreactivity was markedly reduced in RBPMS⁺ RGCs of Cre-injected eyes, verifying effective gene deletion (Fig. 3A, E). p62 and LC3B immunostaining showed higher immunoreactivity in Cre-injected eyes compared with null-injected controls, and colocalization with lysosomal markers was not detected in Cre-treated RGCs. In controls, p62 and LC3B colocalized with lysosomal markers, consistent with intact autophagic flux (Fig. 3B, C, E). Together, the data support the failure of autophagosome formation after the loss of *Atg7*, evidenced by p62 accumulation and elevated LC3B with the absence of lysosomal overlap. At 10 weeks post-injection, immunostaining for 8-hydroxy-2′-deoxyguanosine (8-OHdG) showed markedly elevated oxidative stress in RGCs of Cre-injected eyes relative to null controls, indicating increased oxidative damage following *Atg7* deletion (Figs. 3E and S4A). In the same eyes, COX4 immunostaining revealed increased mitochondrial signal within RGCs, indicative of poor mitochondrial clearance (Fig. 3D, E). In addition, *Atg7* deletion did not alter Rab5 expression, indicating that the early initiation steps of autophagy remain unaffected (Figs. 3E and S4B). In contrast, a marked reduction in Rab7 levels was observed in Cre-injected eyes, suggesting that following the *Atg7*-dependent step, downstream maturation and fusion processes are substantially impaired (Figs. 3E and S4C). Collectively, these findings confirm ATG7 loss in RGCs and support impaired autophagic clearance with oxidative and mitochondrial stress in vivo.

### *Atg5* deficiency phenocopies *Atg7* deletion-induced RGC degeneration 

**Fig. 4:**
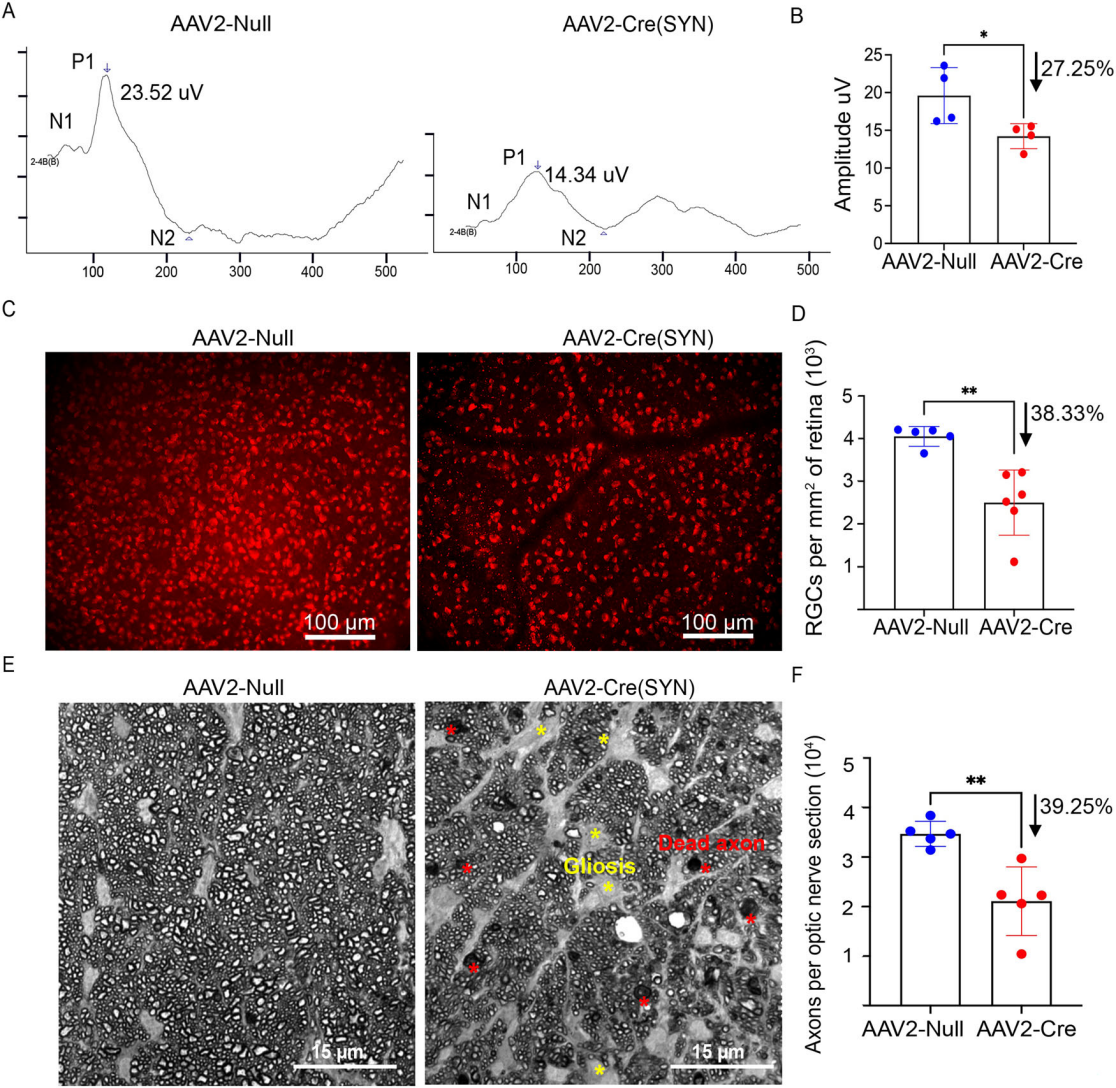
Impaired autophagy leads to functional and structural loss of RGCs and axonal degeneration in *Atg5*^*f/f*^ mice. **A, B** Representative PERG graphs and its analysis showing a significant reduction in the PERG amplitudes in AAV2-Cre(SYN)-injected eyes (*n* = 4 eyes) compared to Null-injected controls (*n* = 4 eyes) after 10 weeks of injection. Statistical test: Unpaired t-test. **C, D** Representative fluorescent microscopic images of retinal flat mounts stained with RBPMS and its analysis showing a significant decrease in RGC counts in AAV2-Cre(SYN) injected eyes after 10 weeks of injection (Null *n* = 5 eyes, Cre *n* = 6 eyes); Scale bar 100 µm. Statistical test: Unpaired t-test. **E, F** Confocal microscopic images of PPD-stained images and their analysis indicating a significant loss of ON axons in Cre-injected eyes (*n* = 5 eyes) compared to null- injected controls (*n* = 5 eyes); Scale bar 15 μm. Statistical test: Unpaired t-test. * represents *p*-value ≤ 0.05 and ** represents *p*-value ≤ 0.01.

To investigate whether the phenotype reflects autophagy deficiency rather than a gene-specific effect, we deleted *Atg5* in RGCs (AAV2-Cre[SYN]) and assessed outcomes 10 weeks post-injection. PERG amplitudes were significantly reduced in Cre-injected eyes, indicating functional loss (Fig. 4A, B). Whole-mount RBPMS labeling showed a 38.33% decrease in total RGC counts versus controls (Fig. 4C, D). PPD staining of the optic nerve revealed a 39.25% reduction in axon number with accompanying degenerative features, including myelin disruption and gliosis (Fig. 4E, F). In addition to these structural and functional outcomes, immunostaining confirmed reduced ATG5 in RBPMS⁺ cells and accumulation of p62, consistent with disrupted autophagic clearance after *Atg5* deletion (Fig. S5A, B). Together with the *Atg7* results, these data support loss of autophagy as the driver of RGC and optic nerve degeneration.

### Defective autophagy leads to neurodegeneration in *Atg7*^*f*/*-*^; Nestin-CreERT2^+^ mice

**Fig. 5:**
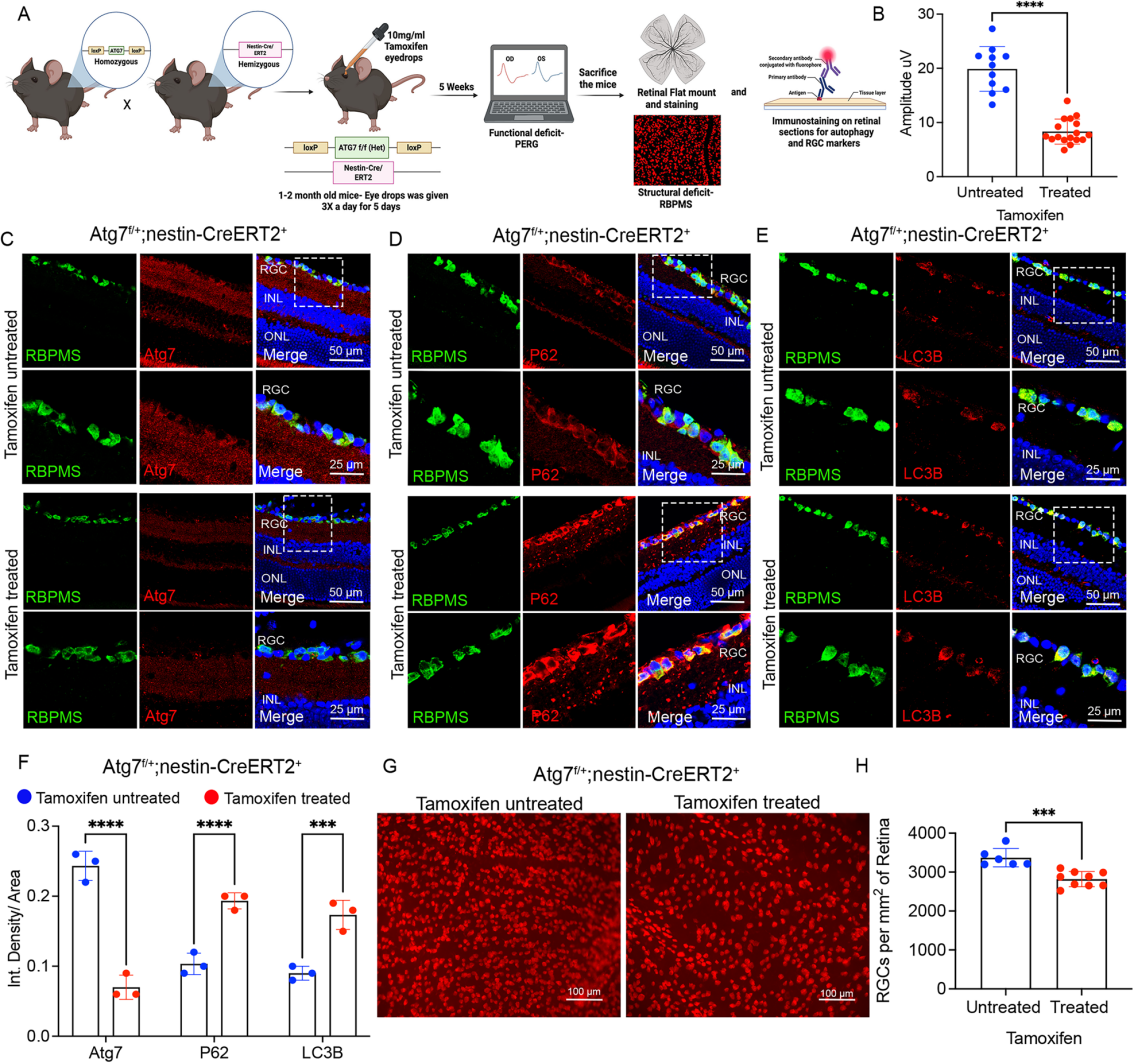
Deficient autophagy in RGCs leads to neurodegeneration in the tamoxifen-induced *Atg7*^*f/-*^; Nestin-CreERT2^+^ mice. **A** Study design illustrating the generation and characterization of impaired autophagy in the *Atg7*^*f/-*^; Nestin-CreERT2 model*.* **B** Significantly reduced PERG amplitudes (Untreated *n* = 11 eyes, treated *n* = 17 eyes) were observed in *Atg7*^*f/-*^*; Nestin-CreERT2*^*+*^ mice treated with tamoxifen compared to untreated control mice. Representative confocal microscopic images of RGCs immunostained for ATG7 (**C**), p62 (**D**), and LC3B (**E**) (red) and costained with RBPMS (green). **F** The bar graph represents the relative fluorescence intensity of the immunostained markers. The intensity for ATG7 was significantly reduced, while the intensity was higher for p62 and LC3B in tamoxifen-treated *Atg7*^*f/-*^*; Nestin-CreERT2*^*+*^ eyes compared to tamoxifen-un-treated controls. The region outlined by the dashed box is shown at higher magnification below each panel. *n* = 3 eyes per group; Scale bar 50 μm; insets 25 μm. Statistical test: Two-way ANOVA with multiple comparisons. **G, H** Representation fluorescent microscopic images stained for RBPMS indicates reduced RGC count in the tamoxifen-treated group (Untreated *n* = 6 eyes, treated *n* = 9 eyes). Scale bar 100 μm. Statistical test: Unpaired t-test. *** represents *p*-value ≤ 0.001. and **** represents *p*-value ≤ 0.0001.

To validate the AAV2-Cre findings with a non-viral approach, *Atg7* floxed mice were crossed with Nestin-CreERT2^+^. Topical tamoxifen (10 mg/mL, 3×/day for 5 days) was administered; tamoxifen-untreated littermates served as controls (Fig. 5A). At 5 weeks post-induction, PERG amplitudes were significantly reduced in tamoxifen-treated *Atg7*^*f/-*^*; Nestin-CreERT2*^*+*^ eyes versus controls (Fig. 5B). Immunostaining of retinal cross-sections confirmed loss of ATG7 (Fig. 5C, F) in RGCs and demonstrated p62 (Fig. 5D, F) accumulation with increased LC3B puncta in tamoxifen-treated mice, consistent with impaired autophagic clearance (Fig. 5E, F). In addition, whole-mount RBPMS staining and quantification showed a concordant decrease in total RGC counts (Fig. 5G, H). Together, the *Atg7*^*f/-*^*; Nestin-CreERT2*^*+*^ model reproduces the functional and structural RGC deficits observed with AAV-mediated *Atg7* deletion, supporting autophagy deficiency as the driver of neurodegeneration.

### Deficient autophagy results in the accumulation of dysfunctional organelles and cellular cargos in the RGC soma

**Fig. 6:**
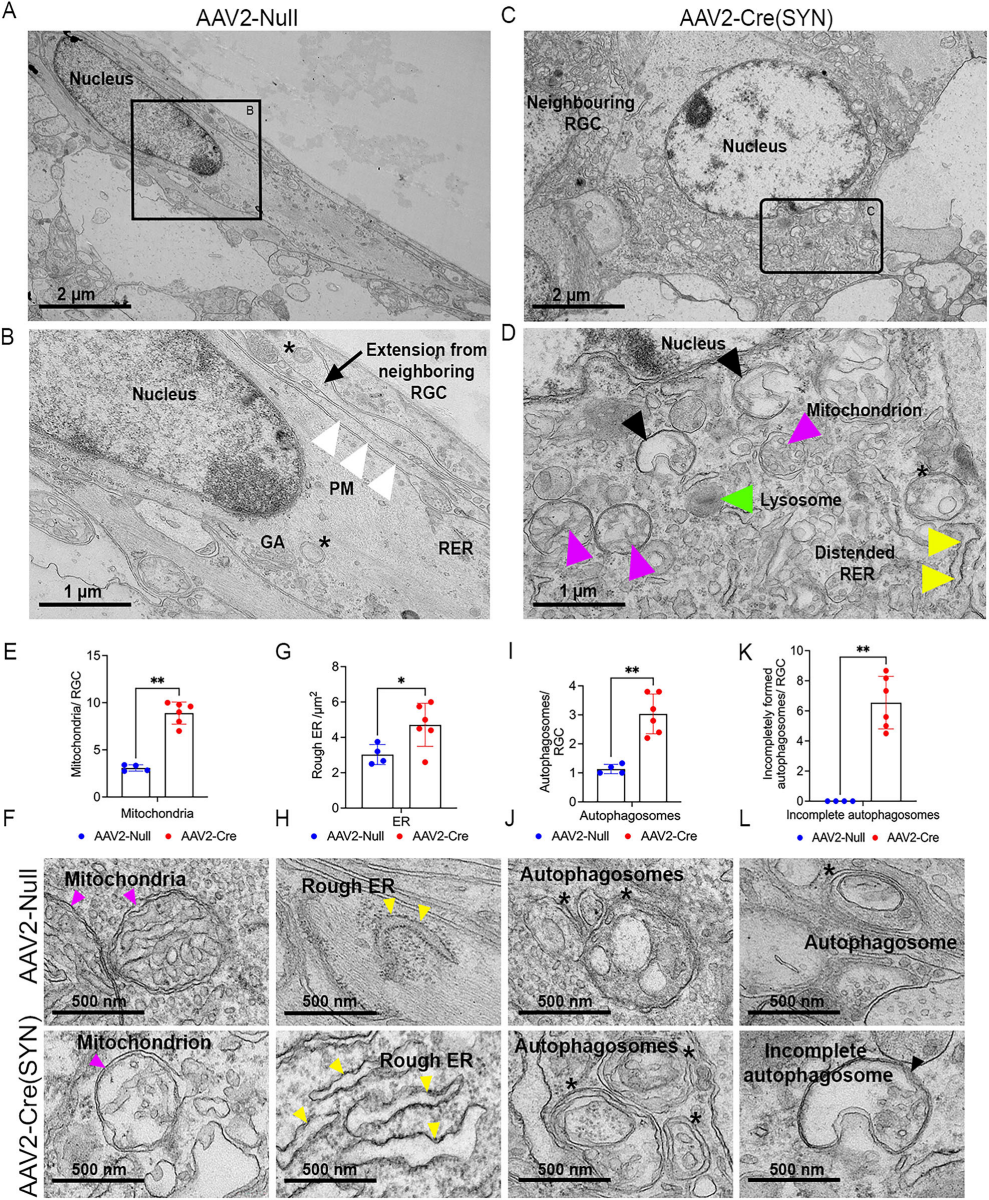
Deficient autophagy leads to the accumulation of damaged organelles and incompletely formed autophagosomes in RGC soma. Representative TEM images of RGC soma after 10 weeks of AAV2-Null (**A, B**) and AAV2-Cre(SYN) injection (**C, D**) showing the accumulation of dysfunctional and impaired organelles in RGC soma in AAV2-Cre-injected *Atg7*^*f/f*^ mice (Null *n* = 4 eyes, Cre *n* = 6 eyes). The inset provides a magnified view of the RGC region, highlighting detailed microstructural features. Quantification of TEM images revealed a significant increase in cellular organelles like mitochondria (**E, F**), RER (**G, H**), autophagosomes (**I, J**), and incompletely formed autophagosomes (**K, L**) in RGCs with impaired autophagy. Statistical test: Unpaired t-test. * represents *p*-value ≤ 0.05 and ** represents *p*-value ≤ 0.01. *Represents autophagosomes; black arrowheads represent incompletely formed autophagosomes, yellow arrowheads represent distended RER, and pink and green arrowheads represent mitochondria and lysosomes, respectively. GA Golgi apparatus, RER Rough endoplasmic reticulum, PM Plasma membrane.

To delineate how deficient autophagy leads to neurodegeneration, we performed transmission electron microscopy (TEM) of AAV2-Null (*n* = 4) and AAV2-Cre(SYN) (*n* = 6) injected eyes 10 weeks post-Cre-injection. Compared to control retinas, which showed normal cellular organelles and structures (Fig. 6A, B), Cre-injected eyes exhibited abnormal accumulation of damaged/dysfunctional organelles, including swollen mitochondria, distended rough and smooth endoplasmic reticulum, fragmented Golgi apparatus with swollen cisternae and synaptic vesicles in the cytoplasm of the RGC soma. In addition, the disintegrated nuclear and cytoplasmic membrane was observed (Fig. 6C–L). Consistent with the loss of ATG7 protein, which is required for the closure of autophagosomes, incompletely formed autophagosomes were observed in AAV2-Cre-injected retinas (Fig. 6C, D, K, L). TEM analysis demonstrated that these autophagosomes were either empty or contained remnants of degraded cytoplasmic material, consistent with an impaired autophagic mechanism. Quantitative analysis from TEM images showed a significant increase in the number of mitochondria (Fig. 6E, F), rough endoplasmic reticulum (RER) (Fig. 6G, H), and autophagosomes (Fig. 6I, J) in autophagy-deficient RGCs compared to controls. In addition, an increase in the number of incompletely formed autophagosomes was evident in autophagy-deficient RGCs (Fig. 6K, L).

Next, we examined whether deficient autophagy leads to axonal degeneration and organelle accumulation in ON axons. TEM analysis of AAV2-Cre injected optic nerves (*n* = 8) demonstrated gliosis, axonal loss, shrunken and dark axoplasm, accumulation of swollen and unhealthy mitochondria and vesicles, degradation and whirling of the myelin sheath and accumulation of cellular debris (Fig. S6A–C). These findings suggest that deficient autophagy impairs the recycling of dysfunctional organelles, leading to their accumulation within the RGC somata and axons.

### Proteomic profiling reveals disrupted organelle homeostasis and metabolic pathways in autophagy-deficient RGCs and optic nerve

*Atg7*^*f/f*^ conditional knockout model exhibits accumulation of dysfunctional organelles within the RGC somata and ON axons. To investigate early molecular changes preceding neurodegeneration, we conducted LC-MS/MS proteomic analysis on retinas and optic nerves from *Atg7*^*f/f*^ mice 2 weeks after AAV2-null or Cre(SYN) injection, aiming to identify differentially regulated pathways in the context of autophagy deficiency. Proteomic analysis revealed 51 upregulated and 31 downregulated proteins (fold change ≥1.5, FDR < 0.05) in Cre-injected retinas compared to the control retinas (Table S2).

**Fig. 7:**
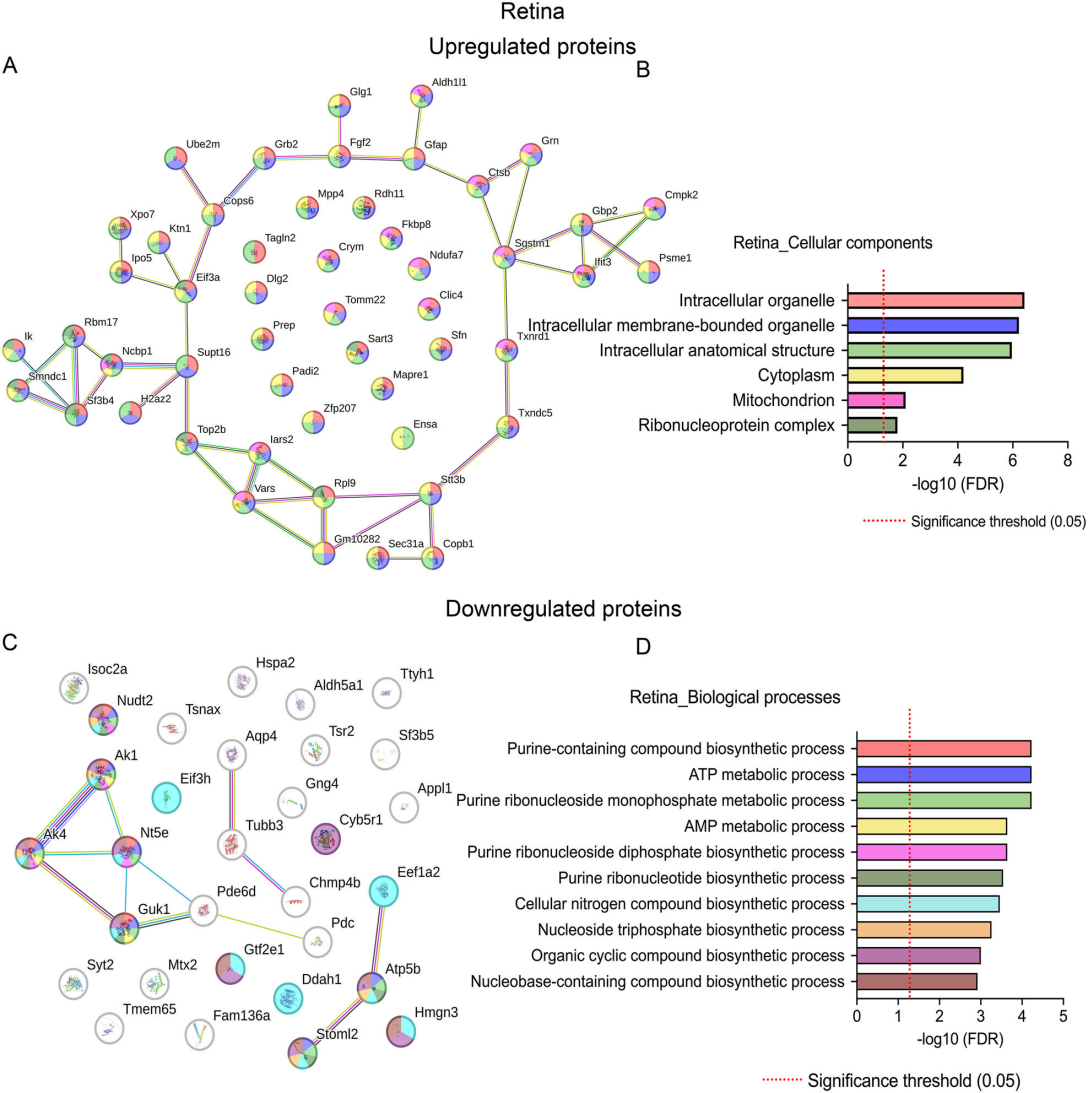
Proteomic analysis of the retina during the earlier stages of impaired autophagy. **A** Functional network map of all proteins upregulated after 2 weeks of AAV2-Cre injection in *Atg7*^*f/f*^ mice. **B** Pathway analysis of proteins mentioned in (**A**) reveals significantly enriched cellular components in the retina. **C** Functional network map of all proteins downregulated after 2 weeks of AAV2-Cre injections. **D** Pathway analysis of proteins in (**C**) reveals significantly enriched biological processes downregulated after the deletion of *Atg7*. *n* = 6 eyes per group; FDR False discovery rate.

We filtered upregulated and downregulated proteins and focused on enriched pathways affected due to impaired clearance mechanisms. The identified protein network was mapped based on functional enrichment, and the biological processes and cellular components were plotted according to log (FDR) (Fig. 7A, B). Analysis of the functional network of the retina revealed upregulation of cellular components, including intracellular organelles (GO:0043229), intracellular membrane-bound organelles (GO:0043231), intracellular anatomical structures (GO:0005622), cytoplasm (GO:0005737), mitochondrion (GO:0005739), and ribonucleoprotein complex (GO:1990904). (Fig. 7B). On the other hand, deficient autophagy downregulated various crucial biosynthetic and metabolic pathways, including purine ribonucleoside monophosphate metabolic pathway (GO:0009167), ATP metabolic pathway (GO:0046034) and purine-containing compound biosynthetic pathway (GO:0072522) (Fig. 7C, D).

Further, analysis of the ON proteomic profile identified 41 upregulated and 8 downregulated proteins (Table S3). Proteomic pathway analysis using the differentially regulated proteins revealed significant enrichment of cellular components, including cytoplasm (GO:0005737), intracellular membrane-bound organelles (GO:0043231), intracellular organelles (GO:0043229), and intracellular anatomical structures (GO:0005622) (Fig. S7A, B). Gene ontology analysis further revealed the downregulation of protein networks related to cytoskeletal structure (TUBA4A, TUBB5), mitochondrial processes (HspD1 and SOD2), and cellular repair (PCNA) pathways. (Fig. S7C). Notably, proteomic data support ultrastructural findings from TEM, highlighting the critical role of autophagy in maintaining the balance between cellular integrity and metabolic homeostasis in RGCs and the optic nerve.

## Discussion

RGCs relay immense visual information from the retina to the brain, requiring active transport and constant turnover of proteins, macromolecules, and organelles [23]. As long-lived and postmitotic neurons, RGCs are particularly vulnerable to the buildup of misfolded protein aggregates and damaged organelles, which are typically diluted through cell division in proliferating cells [24]. Autophagy plays a vital role in degrading and recycling these cellular components to preserve neuronal homeostasis [25–27]. However, the precise physiological role of constitutive autophagy in RGCs remains largely uncharacterized. We demonstrate that constitutive autophagy is critical for RGC function and survival. RGCs display robust basal autophagy under physiological conditions, and disruption of autophagy in RGCs via conditional knockout of *Atg7* or *Atg5* results in progressive RGC degeneration. Notably, impaired autophagy hampers the clearance of dysfunctional organelles, causing their accumulation in the soma and axons of RGCs. Proteomic analysis further corroborated these findings and revealed an enrichment of cellular components, including the cytoplasm, membrane-bound organelles, mitochondria, and structural elements. Together, our studies indicate that functional autophagy is required for RGC function and survival, and deficient autophagy leads to the accumulation of organelles and neurodegeneration.

In this study, we used three well-established autophagy models: floxed *Atg7*, floxed *Atg5*, and *Atg7*^*f/-*^*; Nestin-CreERT2*^*+*^ mice for tamoxifen-inducible deletion [10, 28, 29]. To delete genes in adult RGCs, we delivered Cre with AAV2 under either CMV or SYN promoters, leveraging AAV2’s RGC tropism [5]. CMV drove strong Cre expression but displayed broader retinal tropism with rare INL labeling (Fig. S1A), so we performed the main experiments with SYN, which preferentially targets RGCs [5, 12]. Although the onset with SYN was slightly later than CMV, it provided the required cell-type specificity. To complement the viral approach and add temporal control, we also used *Atg7*^*f/-*^*; Nestin-CreERT2*^*+*^ mice, which enable tamoxifen-inducible gene deletion in neurons [11] (including, but not limited to, RGCs). Importantly, the onset and magnitude of the neurodegenerative phenotype varied with the promoter (CMV vs. SYN), the autophagy gene targeted (*Atg7* vs*. Atg5*), and the Cre- delivery strategy (AAV vs. Nestin-CreERT2); accordingly, assay time points in this study were aligned to each model’s kinetics. To achieve non-viral, germline recombination that is RGC-restricted, we will employ the Chrnb3-Cre BAC driver [30] in future studies, in contrast to Nestin-CreERT2, which is not RGC-specific. Together, these viral and genetic tools provide a robust framework to interrogate autophagy in RGCs.

RGC degeneration is a hallmark of primary open-angle glaucoma (POAG), an ocular disease characterized by elevated intraocular pressure (IOP) and progressive vision loss [31, 32]. However, the molecular mechanisms underlying RGC degeneration in POAG remain poorly understood. Autophagy has been implicated in both protective and pathological roles, depending on experimental context or disease model [12–17]. In acute injury settings like ON axotomy, autophagy appeared beneficial where pharmacological activation improved RGC survival, while deletion of *Atg5* or *Atg4b* accelerated cell loss [12]. In contrast, studies in chronic glaucoma models, such as *DBA/2J-Atg4b*^*ko*^ or *TGFβ2*-induced ocular hypertension, reported that autophagy deficiency reduced IOP and RGC degeneration [16], suggesting autophagy might contribute to disease progression under sustained stress. While informative, these models rely on either non-essential autophagy genes or lack cell-type specificity, limiting mechanistic clarity. In addition, the global *Atg4B* knockout mice are not ideal for studying tissue-specific autophagy. *Atg4B* knockout mice exhibit developmental defects, including growth retardation, metabolic defects, and neurological impairments from birth [33–35]. Global *Atg4B* knockout mice did not show RGC functional or structural loss, likely due to compensation by other *Atg4* paralogs (e.g., *Atg4A, Atg4C, Atg4D*), masking specific phenotypes [36–38]. In addition, other compensatory pathways might obscure tissue-specific functions. For example, alternative degradation pathways (e.g., the ubiquitin-proteasome system) may be upregulated, leading to misleading interpretations [39]. Importantly, none of these studies investigated the basal role of autophagy in RGCs by selectively knocking out key autophagy-related genes. Our study overcomes these limitations by conditionally deleting the core autophagy genes *Atg5* and *Atg7*, with a targeted deletion in RGCs using AAV2-Cre and complementary validation using the *Nestin-CreERT2*^+^ system. Importantly, we assessed autophagy function in the absence of injury or IOP elevation, allowing us to isolate its basal, homeostatic role. Our studies demonstrate that autophagy is actively maintained under normal conditions and is essential for RGC integrity, as evidenced by the loss of key autophagy genes alone, which is sufficient to cause progressive soma and axonal degeneration, organelle accumulation, and visual dysfunction. These findings establish a direct, cell-autonomous requirement for autophagy in RGC survival and highlight its foundational role beyond stress-induced response.

In our models, loss of *Atg7* in RGCs produced a molecular signature of impaired autophagic clearance. p62 accumulated, and LC3B immunoreactivity increased; in line with prior work showing recruitment of endogenous LC3-I to p62/ubiquitin-positive aggregates when autophagy is compromised [29, 40, 41], this pattern is best interpreted as stalled clearance rather than increased autophagosome biogenesis [42, 43]. This interpretation is supported by the absence of p62- or LC3B-LAMP1 colocalization in Cre-treated RGCs (Fig. 3B, C, E), in contrast to the overlap seen in controls, and by unchanged Rab5 (Fig. S4B) with reduced Rab7 (Fig. S4C), indicating a defect at late maturation/fusion steps [44, 45]. We also observed higher mitochondrial markers (COX4 (Fig. 3D, E); TOM20, data not shown) together with elevated 8-OHdG, consistent with defective mitophagy and oxidative damage (Fig. S4A). Taken together and in agreement with reports that autophagy deficiency drives accumulation of protein aggregates and damaged mitochondria [25, 39–41], these findings support a model in which constitutive autophagy maintains proteostasis and organelle quality control in RGCs; when this pathway is disrupted, undegraded cargo and mitochondria accumulate, oxidative stress rises, and neurodegenerative changes ensue.

Strikingly, several cellular defects arising from autophagy deficiency mirror hallmark features observed in the early stages of POAG. Notably, the cellular abnormalities observed in our autophagy-deficient models, like mitochondrial accumulation, synaptic vesicle buildup, and protein aggregates, closely resemble early pathological features reported in POAG. Mitochondrial dysfunction and synaptic remodeling have been linked to RGC vulnerability in glaucoma [26, 46, 47]. These changes, occurring in the absence of elevated IOP or injury, suggest that impaired autophagy is sufficient to initiate stress-related phenotypes characteristic of RGC degeneration. This highlights the role of autophagy as a critical homeostatic mechanism, whose disruption may potentially contribute to early events in glaucomatous RGC loss.

TEM analysis of the RGC soma revealed a buildup of damaged mitochondria, swollen rough ER, and numerous incomplete, cup-shaped autophagosomal structures (Fig. 6C–L). These immature autophagosomes resembled early phagophores and appeared to originate from ER membranes, a known source of autophagosome initiation [48, 49]. Although some mature autophagosomes and autolysosomes were also observed, the degradation process appeared inefficient. These structures may represent preexisting autophagosomes or reflect partial compensation by Rab-dependent, noncanonical autophagy pathways [20, 50, 51], which appear insufficient to restore normal clearance in the absence of essential autophagy regulators like *Atg7*.

RGCs possess long, polarized axons that depend on tightly regulated intracellular trafficking to maintain homeostasis. In neurons, autophagy is a compartmentalized process where autophagosomes are often formed in distal axons, mature locally into autolysosomes, and are transported to the soma for degradation [52, 53]. Since axons have limited degradative capacity, damaged mitochondria and proteins must be trafficked efficiently for clearance. Disruption of this system may compromise both axonal integrity and soma viability by promoting the accumulation of undegraded material and preventing the delivery of essential components needed for maintenance and function at distal neuronal sites [54, 55]. In our mouse models, we observed functional decline and axonal degeneration. This disruption is supported by our observations of accumulated damaged mitochondria, vesicles, and immature autophagic structures in RGC axons and soma, indicating a failure in effective cargo processing and turnover. A previous study in Purkinje neurons similarly demonstrated that *Atg7* is critical for membrane trafficking and protein degradation, and its deletion resulted in axonal swelling and dystrophy [29]. These findings suggest that *Atg7* loss in RGCs disrupts autophagy-associated trafficking processes, potentially affecting both anterograde and retrograde transport, leading to the buildup of undegraded material. Thus, autophagy may serve as a degradative pathway and a key regulator of intracellular transport and recycling in long-projection neurons like RGCs.

To complement our ultrastructural and functional analyses, we profiled the retinal and optic nerve proteomes to examine the molecular consequences of autophagy deficiency in RGCs (Figs. 7 and S7). In *Atg7*-deficient retinas, differentially expressed proteins were enriched in mitochondria, cytoplasm, membrane-bound organelles, and ribonucleoprotein complexes, consistent with TEM evidence of organelle accumulation and impaired clearance. We also observed a coordinated downregulation of biosynthetic and metabolic programs, including pathways related to mitochondrial function, cytoskeletal dynamics, and cellular repair, suggesting a systems-level shift away from anabolism under autophagy loss. We interpret these proteome-wide shifts as associations rather than demonstrated causality; a plausible model is that impaired autophagy/mitophagy reduces organelle quality control and elevates proteotoxic/oxidative stress [supported by increased 8-OHdG (Fig. S4A)], which secondarily constrains metabolic and translational capacity in RGCs. Gene ontology analysis of the optic nerve proteome further underscores axonal vulnerability, where efficient turnover of cytoskeletal and mitochondrial components is essential for long-range transport and survival. Ongoing studies using RGC-restricted flux reporters and direct mitochondrial/metabolic assays (e.g., ATP content, membrane potential, metabolic flux) are being carried out to test this model. Collectively, these proteomic data align with the structural and cellular degeneration observed in autophagy-deficient RGCs and highlight candidate pathways for future therapeutic targeting [56, 57].

While our focus is on RGC-targeted functional and structural assays, several boundaries of the current work are worth noting. We evaluated both AAV2-Cre(CMV) and AAV2-Cre(SYN) in pilot experiments; because CMV labeled RGCs together with a subset of inner retinal neurons, we prioritized SYN for cell-type focused analyses despite its later phenotype onset. In vivo bafilomycin supports basal autophagy activity in the ganglion cell layer, but we did not perform RGC-enriched LC3-I/LC3-II immunoblotting or deploy genetic flux reporters at RGC resolution. The study is RGC-centric and does not quantify autophagy in other retinal layers (e.g., RPE). We also did not directly assess mitochondrial bioenergetics (e.g., ATP production or mitochondrial membrane potential) in RGCs. We are actively addressing these points in ongoing work and will incorporate them in future publications.

In conclusion, our findings show that RGCs exhibit robust constitutive autophagy, which is critical for neuronal function and survival. Loss of autophagy in RGCs leads to neurodegeneration, marked by the accumulation of defective organelles, vesicles, and proteins within the soma and axons. Our proteomic analysis further supports these observations by revealing the enrichment of cellular components related to membrane-bound organelles, cytoplasm, mitochondria, and structural elements, highlighting the widespread cellular disruption caused by impaired autophagy. Importantly, these findings provide valuable insight into the physiological role of autophagy in RGCs and shed light on the potential pathological mechanisms of POAG. Understanding the role of autophagy in RGC health could offer new perspectives on disease progression and potential therapeutic targets.

## Methods

### Antibodies and reagents

Details of antibodies and reagents used in this study are listed in Table S1.

### Animals

C57BL/6 J (Strain ID# 000664), *Atg7*^*f/f*^ mice (Strain ID# 034429), and Ai14 mice expressing robust tdTomato (Strain ID# 007914) were obtained from the Jackson Laboratories (Bar Harbor, ME, USA). *Atg5*^*f/f*^ (Strain ID# RBRC02975) mice were generously provided by Nobru Mizushima (The University of Tokyo, Tokyo, Japan), and these animals were obtained from Thomas Ferguson’s lab (Washington University, School of Medicine, St. Louis, Missouri, USA). *Nestin-Cre-ERT2*^*+*^ transgenic mice were purchased from the Jackson laboratory (Strain ID# 016261) and were bred to *Atg7* ^*f/f*^ mice to obtain *Atg7*^*f/-*^ harboring Nestin-Cre-ERT2^*+*^. Animals were housed and bred in standard 12-hour light/12-hour dark conditions. Animals were fed standard chow *ad libitum* and kept in cages with dry bedding. Unless otherwise specified, mice were 12 weeks of age at the time of experimentation, whereas *Atg7*^*f/-*^; Nestin-Cre-ERT2^+^ animals received tamoxifen eye-drop treatment at 1–2 months of age.

### Animal ethics and study approval

Vertebrate animals were included in the study. All the animal experiments were carried out in accordance with the guidelines of the Association for Research in Vision and Ophthalmology (ARVO) statement on the use of animals in ophthalmic and vision research. Experimental protocols involving mice were approved by appropriate IACUC and biosafety committees at the University of California, Irvine (*Protocol numbers: AUP-22-159 and AUP-25-158*). Animal euthanization was carried out via inhalation of carbon dioxide followed by cervical dislocation, as approved by institutional IACUC protocol. This study did not involve human participants; therefore, informed consent to participate was not applicable, and no identifiable human images are included in this manuscript.

### Intravitreal injections

RGC-specific expression of Cre-recombinase was achieved using intravitreal (IVT) injection of AAV2 under the control of CMV and SYN promoters as described previously [22, 58, 59]. AAV2 expressing an empty cassette or Cre was purchased from Signagen Laboratories (Gaithersburg, MD). Briefly, 12-week-old mice were anesthetized and bilaterally injected with 2 μl of AAV2-Null, AAV2- Cre(CMV), or AAV2-Cre(SYN) (1 × 10^3^ vg/ml) in the vitreous chamber through the sclera using a Hamilton syringe fitted with a sterile 33-gauge needle. Animals that developed ocular abnormalities after intravitreal injection (e.g., corneal opacity, cataract, or signs of intraocular inflammation) were excluded from functional and histological analyses, because these changes preclude reliable PERG recording and retinal/ON assessment.

### Animal allocation and selection criteria for downstream assays

Animals were assigned to groups on the basis of genotype and viral construct (AAV2-Cre vs AAV2-null); no additional formal randomization procedure was used. For downstream structural and functional analyses (RBPMS immunostaining, immunohistochemistry, TEM, mass spectrometry), we selected a subset of AAV2-Cre injected eyes that showed the expected reduction in PERG amplitude relative to control eyes. These eyes were then allocated to the different downstream assays according to experimental needs, without additional formal randomization.

### Bafilomycin A1 treatment to assess in vivo autophagic flux

To assess autophagic flux, twelve-week-old C57BL/6 mice were anesthetized with ketamine/xylazine (100/10 mg /kg). Topical proparacaine was applied, and 0.3% hypromellose gel was used to prevent corneal drying. Bafilomycin A1 (5 µM; 2 µL/eye) was delivered by bilateral intravitreal injection; DMSO vehicle served as control. Mice were euthanized 2 h post-injection; eyes were enucleated and processed for cryosectioning and immunostaining.

### Pattern electroretinogram (PERG)

The function of RGCs was evaluated via PERG using the Celeris system (Diagnosys LLC, UK) as per the manufacturer’s protocol. Briefly, the animals were dark-adapted overnight before performing PERG. The mice were then anesthetized intraperitoneally with ketamine/ xylazine (100/10 mg /kg), and the pupils were dilated with 1% tropicamide (Somerset Therapeutics, Hollywood, FL). To avoid corneal dryness, a moisturizing gel containing 0.3% hypromellose (Alcon, Fort Worth, TX) was applied to the eyes. The animals were placed on an in-built heating pad throughout the recording session to maintain body temperature at 37 °C. A patterned stimulus presenting a black-and-white grating at a spatial frequency was used to induce an RGC response in a dark-adapted mouse. The parameters to measure the PERG amplitude were set to a spatial frequency of 0.155 cycles/degree, temporal frequency of 2.1 reversals/sec, contrast of 100%, and substantial averaging (200–400 sweeps). The data were analyzed using the Espion V6 (Diagnosys LLC, UK) software, and the P1 (P1-N2) peak was considered PERG amplitude.

### Optical coherence tomography

Spectral-domain optical coherence tomography (SD-OCT) was used to image and analyze the retinal nerve fiber layer (RNFL) non-invasively. The SD-OCT images were taken using the Envisu RR210-HR-SD-OCT (Bioptigen, Durham, NC). The mice were anesthetized using ketamine/ xylazine (100/10 mg/kg). The pupils were dilated using 1% tropicamide and 2.5% phenylephrine. To maintain moisture and clarity of the cornea, 0.3% hypromellose was applied topically. The mouse was positioned on the imaging platform, and the images were acquired in radial volume with the following parameters: A-Scans/ B-Scans = 1000 line, B-Scans/ Volume = 1000 scans, Frames/ B-Scan = 1 frame, Inactive A-Scans/B-Scan = 80 lines and number of volumes = 1 at a diameter of 1.4 mm. RNFL thickness was determined using the built-in Bioptigen Envisu analysis software.

### Mouse eye and ON dissection

Mice were euthanized and dissected in accordance with the detailed protocol provided in the Supplementary Information.

### Tissue processing, cryo-sectioning, and immunostaining

Eyes were enucleated and processed following the detailed protocol described in the Supplementary Information.

### Confocal microscopy and mean fluorescence intensity measurement

Refer to the supplementary information.

### Retinal flat mount preparation and RBPMS staining

To analyze the structural loss of RGCs, flat mount retinal staining with the RNA-binding protein with multiple splicing (RBPMS) antibody was performed as described previously [60, 61]. The enucleated eyes were fixed briefly with 4% PFA for 2 h at room temperature. The eyes were then washed thrice with 1X PBS to remove residual PFA. The anterior segments were dissected, and the lens was gently pushed out. The neural retina was separated from the posterior segment and washed thoroughly with 1X PBS containing 0.3% Triton X-100 for one hour with gentle agitation. The retinas were then incubated with a blocking solution containing 10% goat serum and 0.3% Triton × 100 for 1 h at room temperature. Following blocking, the isolated retinas were incubated with RBPMS antibody for 3 days at 4 °C. The retinas were subsequently incubated with the corresponding secondary antibody (Supplementary Table 1) for 2 h at room temperature and then mounted with a mounting medium. Between the steps, the retinas were washed several times with 1X PBS for 2 h at room temperature with gentle agitation. Retinal flat mounts were imaged at 20× magnification using a Keyence fluorescence microscope (Keyence Corporation, Itasca, IL). For quantitative analysis, each retina was divided into four quadrants, and six non-overlapping fields were imaged per quadrant (24 fields/retina total) using identical acquisition settings across groups. Fields were selected using a predefined quadrant-based sampling scheme to provide unbiased, representative coverage across the retina, and selection was not restricted to the injection site, as the goal was to assess overall retinal effects rather than localized changes. Imaging and quantification were performed in a masked manner, and the number of RGCs per image was counted in ImageJ. The numbers are further extrapolated to represent the RGC count per 1 mm^2^ of the retina.

### PPD staining

Paraphenylenediamine (PPD) staining of the ON was performed to determine ON degeneration as described previously [60, 61]. Briefly, the optic nerves were dissected from the eye and fixed with 3% glutaraldehyde/ 3% PFA prepared in 1X PBS overnight at 4 °C. Subsequently, the optic nerves were treated in 1% osmium tetroxide at 4 °C, followed by washes with 0.1 M phosphate buffer and 0.1 M sodium acetate buffer. The optic nerves were dehydrated in graded ethanol concentrations and embedded in resin (Eponate-12; Ted Pella). Transverse semi-thin sections (1 μm) were cut and stained with 1% PPD for 10 min. Five non-overlapping confocal microscopic images of the ON section were captured at 100X magnification. The number of axons was manually counted using ImageJ software. The number of axons counted in a specific area (20 μm × 20 μm) was extrapolated to the total ON cross-section area and represented graphically.

### Transmission electron microscopy

To study the ultrastructural details of the RGCs and optic nerve, transmission electron microscopy (TEM) was performed as described previously [60, 61]. Briefly, the eyes and optic nerves were dissected following cardiac perfusion fixation in 4% PFA. The tissues were further fixed in 1.6% PFA and 1.5% glutaraldehyde in 0.1 M cacodylate buffer (pH 7.2) for 48 h. The tissues were then incubated with 1% osmium tetroxide in a 100 mM cacodylate buffer, followed by aldehyde fixation. After ethanol dehydration, the tissues were embedded in an Eponate 812 resin. 1 μm ultra-thin sections were cut using an ultramicrotome (EM UC6; Leica) and stained with uranyl acetate and Reynolds lead citrate. Images were captured using a Hitachi H-7800 TEM equipped with an AMT NanoSprint15 high-resolution, high-sensitivity camera system with a 5056 ×2960-pixel sensor in the Central Microscopy Research Facility at the University of Iowa. For organelle quantification, a minimum of 5 and up to 10 RGCs were analyzed per sample. Quantification was performed using ImageJ software, either on a per-cell basis or normalized per 1 μm² area, depending on the abundance and distribution of the specific organelle being assessed.

### Protein sample preparation

Freshly dissected mouse retinas and optic nerves were harvested from the eye cups and suspended in urea buffer containing 8 M Urea, 0.1 M Tris-HCl (pH 8.5), and a protease inhibitor cocktail. The suspension was sonicated on ice for 10 min, followed by centrifugation at 12,000 × *g* for 10 min at 4 °C, and the supernatant was collected. Protein concentration was measured using nanodrop, and 30 kDa protein was calculated as the ideal concentration for this protocol. 50 mM ammonium bicarbonate was added to each sample to decrease the urea concentration. The proteins were then reduced with 10 mM DTT (Dithiothreitol) for 1 hr at 56 °C and alkylated with 20 mM iodoacetic acid for 30 min at room temperature in the dark. Free trypsin was added to the protein solution at a trypsin-to-protein ratio 1:50 and incubated overnight at 37 °C. The tryptic activity was quenched by 0.1% trifluoracetic acid, and the samples were added to an activated Sea-Pak column. The proteins were eluted using an elution buffer (80% acetonitrile and 0.1% TFA) and collected in a fresh tube. The samples were dried in a speed vacuum at 45 C for 1 h and were diluted in a loading buffer.

### Mass spectrometry

Proteomic profiling was conducted using an UltiMate 3000 UHPLC system coupled to an Orbitrap Fusion Lumos mass spectrometer (Thermo Fisher Scientific) with an ESI nanospray source. Peptides were separated on an Acclaim PepMap RSLC column (50 cm × 75 μm) using a 57-minute gradient (4–25% ACN with 0.1% FA) at a flow rate of 300 nL/min. MS1 scans were acquired in the Orbitrap (375–1800 m/z) with an AGC target of 8 × 10⁵ and 50 ms injection time. MS/MS scans were acquired in top-speed data-dependent mode (3 s cycle), using stepped HCD (NCE 20 ± 5%) with an AGC target of 1 × 10⁴ and 35 ms injection time. Dynamic exclusion was set at 30 s.

### Label-free quantification analysis

Raw MS data were processed using MaxQuant (v1.5.2.8) with peptide identification against the UniProt mouse database. The search parameters included an initial precursor mass tolerance of 20 ppm and a fragment mass tolerance of 0.5 Da. Up to two missed cleavages were allowed for trypsin digestion. Carbamidomethylation of cysteine was set as a fixed modification, and methionine oxidation was considered a variable modification. A 1% false discovery rate (FDR) was applied to filter peptide identifications.

### Blinding

Investigators performing the in vivo procedures (intravitreal injections, tamoxifen treatments, PERG recordings) were aware of the genotype and viral construct (Cre vs null). For outcome quantification, all images used for RBPMS⁺ cell counts and fluorescence intensity measurements were coded and analyzed by observers blinded to genotype and treatment.

### Statistics

No formal statistical method was used to predetermine sample size. Group sizes were based on our previous experience with similar retinal and ON experiments and on published studies in the field and were chosen to detect biologically meaningful effects while minimizing animal use. Data are presented as mean SD, and the number of eyes (n) is reported in each Fig. legend. Statistical analyses were performed using Prism 10.0 software (GraphPad Software, San Diego, CA). Comparisons between two groups were performed using unpaired two-tailed Student’s *t*-tests. For experiments involving three or more groups or factors, one-way or two-way ANOVA with appropriate multiple-comparison tests was used, as indicated in the Fig. legends. Data distributions and variability were inspected visually and were considered compatible with the assumptions of these tests (approximately symmetric distributions and comparable variances across groups). A *p* value < 0.05 was considered statistically significant.

## Supplementary information

Supplemental Material

## Data Availability

All data supporting this study are included in the manuscript and its accompanying supplementary materials.
