# Supplementary material for: Deficient autophagy in retinal ganglion cells impairs the degradation of intracellular organelles, leading to neurodegeneration
Source: Cell Death Dis. 2026 May 30;17(1):677. doi: 10.1038/s41419-026-08927-3 (PMC13434108; doi:10.1038/s41419-026-08927-3)
Supplement: Supplementary file 1 [file 41419_2026_8927_MOESM1_ESM.pdf]

Supplementary Information

Methods

Mouse eye and optic nerve dissection. The mice were euthanized via carbon dioxide inhalation followed by cervical dislocation. The animals were then decapitated, and the skull was exposed by making a sagittal cut from the base of the skull to the snout. Using appropriate spring scissors, the skull cap was dissected from the parietal region to the nasal portion, exposing the brain. The brain was lifted to cut the optic nerve at the optic chiasm, thereby disconnecting the nerves from the brain. The eyes were carefully separated from the surrounding bones, muscles, and connective tissues. After dissecting the eye, the remnants of extraocular muscles and connective tissues attached to the eyes and optic nerve were cleared under a dissection microscope (Nikon SMZ745, Tokyo, Japan).

Tissue processing, cryo-sectioning, and immunostaining. Eyes were enucleated and fixed in 4% paraformaldehyde (PFA) for 2 hours at room temperature. The eyes were then incubated in increasing concentrations of sucrose (overnight in 10%, 20%, and 30% sucrose, respectively) at 4^0C. The eyes were washed with 1x PBS and embedded in optimal tissue cutting temperature (OCT) media (Sakura Finetek, Torrance, CA) using tissue molds. 10 μm thick cross sections were cut using the cryostat (Leica M1860, Wetzlar, Germany). The OCT-embedded sections were incubated at 60^0 C for 10 minutes and washed with 1X PBS. The tissue sections were blocked with blocking solution (10% goat serum and 0.2% Triton X 100 in 1XPBS) for 2 hours at room temperature. Primary antibodies (Supplementary Table 1) were added to the slides and incubated overnight at 4^0C. The next day, the sections were incubated with appropriate secondary antibodies for 2 hours at room temperature. The slides were washed thrice with 1X PBS, mounted, and sealed using mounting media containing nuclear counterstain DAPI (Vectashield, Newark, CA). Sections incubated without primary antibodies were used as negative controls. The images were captured using a laser scanning confocal microscope (Leica SP8, Wetzlar, Germany).

Confocal microscopy and mean fluorescence intensity measurement. Fluorescent images of immunostained retinal sections were acquired using a Leica SP8 laser scanning confocal microscope (Leica Microsystems, Germany). Imaging parameters were set as follows: Alexa Fluor 488 was excited with a 488 nm blue laser and detected within an emission bandwidth of 496-530 nm; Alexa Fluor 568 was excited with a 552 nm green laser and detected within 550-600 nm; and DAPI was excited using a 350 nm UV laser, with emission collected between 340-400 nm. To measure the mean fluorescence intensity for each immunostained marker, we analyzed three non-overlapping sections

from each eye. Within each section, defined regions of interest (ROIs) were manually selected using the Leica SP8 confocal microscopy software. ROIs were drawn over the RGC layer and other relevant retinal layers to measure fluorescence intensity in the appropriate channels corresponding to each fluorophore. Identical acquisition settings (laser power, gain, and offset) were maintained across all samples to ensure consistency. The mean intensity values were then calculated and used for quantitative comparison in RGCs and between retinal layers.

Supplementary Tables

Table S1. Details of antibodies, viral vectors, and reagents used in this study.

Primary Antibody	Source	Catalogue No.	Dilution
ATG7	Thermo Scientific	PA5-35203	1:200
ATG5	Novus Biologicals	NB110-53818	1:100
P62	MBL International	PM045	1:200
LC3B	Millipore Sigma	L7543	1:200
COX4	Proteintech	11242-1-AP	1:200
Tubulin β III	Abcam	1063373-11	1:250
RBPMS	Genetex	GTX118619	1:400
RBPMS	Thermo Scientific	PA5-119676	1:500
Lamp1	Cell Signaling Technology	15665S	1:100
Rab5	Santa Cruz	sc-46692	1:50
Rab7	Santa Cruz	sc-376362	1:50
**Secondary Antibody**	**Source**	**Catalogue No.**	**Dilution**
Goat anti-rabbit 568	Invitrogen	A11011	1:500
Goat anti-rabbit 488	Invitrogen	A11034	1:500
Goat anti-mouse 568	Invitrogen	A110046	1:500
Goat anti-mouse 488	Invitrogen	A11001	1:500

Viral Vectors	Source	Catalogue No.
AAV2-NULL	Signagen laboratories	SL100811
AAV2-CMV-Cre	Signagen laboratories	SL100813
AAV2-SYN-NULL	Signagen laboratories	SL116349
AAV2-SYN-Cre	Signagen laboratories	SL101462

**Reagents**	**Source**	**Catalogue No.**
Bafilomycin A1	Cell Signaling Technology	54645S
1% Tropicamide	Somerset Pharma LLC	70069-121-01
Night gel (0.3% hypromellose)	Systane, Alcon	25368501-0519
Antifade mounting media with DAPI	Vectashield	H-1200
D-Sucrose	Fischer bioreagents	BP220-1
Triton X 100	Millipore Sigma	T8787-100ml
Tamoxifen	Millipore Sigma	T2859-1g
Corn oil	Millipore Sigma	C8267-500ml
Goat Serum	Millipore Sigma	S26-Liter
PBS Tablets	Gibco	18912-014
Paraformaldehyde 16%	Electron Microscopy Sciences	15710
Glutaraldehyde 8%	Electron Microscopy Sciences	16020
Sodium Cacodylate	Electron Microscopy Sciences	11653
Osmium Tetroxide	Electron Microscopy Sciences	19100
Eponate 812	Electron Microscopy Sciences	14120
Eponate-12	Ted pella	18010

Table S2. Differentially regulated proteins in retina 2 weeks post Cre injection.

**Retina_Upregulated Proteins**				
**Uniprot accession number**	**Gene ID**	**Protein Names**	**log2FC**	**-log10(p)**
Q3UPL0	SC31A	Protein transport protein Sec31A	3.00	1.53
Q8BKC5	IPO5	Importin-5	3.00	2.00
Q3U5Q7	CMPK2	UMP-CMP kinase 2, mitochondrial	2.90	2.51
Q91XM9	DLG2	Disks large homolog 2	2.81	2.15
Q8R0Y6	AL1L1	Cytosolic 10-formyltetrahydrofolate dehydrogenase	2.66	1.44
Q9JLI8	SART3	Squamous cell carcinoma antigen recognized by T-cells 3	2.57	1.94
P51410	RL9	Large ribosomal subunit protein uL6	2.55	1.56
P97371	PSME1	Proteasome activator complex subunit 1	2.41	2.05

Q9JMH6	TRXR1	Thioredoxin reductase 1, cytoplasmic	2.23	1.51
O88545	CSN6	COP9 signalosome complex subunit 6	2.15	2.02
Q08642	PADI2	Protein-arginine deiminase type-2	2.08	1.36
Q64345	IFIT3	Interferon-induced protein with tetratricopeptide repeats 3	1.96	1.62
Q9QYB1	CLIC4	Chloride intracellular channel protein 4	1.95	1.65
Q64337	SQSTM	Sequestosome-1	1.95	2.08
P15655	FGF2	Fibroblast growth factor 2	1.91	2.27
Q9WVA4	TAGL2	Transgelin-2	1.87	1.62
Q9Z1M8	RED	Protein Red	1.86	1.47
Q9Z0E6	GBP2	Guanylate-binding protein 2	1.67	1.96
Q3TDQ1	STT3B	Dolichyl-diphosphooligosaccharide--protein glycosyltransferase subunit STT3B	1.62	1.40
P10605	CATB	Cathepsin B	1.62	1.55
P23116	EIF3A	Eukaryotic translation initiation factor 3 subunit A	1.59	2.75
Q3UYV9	NCBP1	Nuclear cap-binding protein subunit 1	1.57	1.36
P28798	GRN	Progranulin	1.52	2.97
Q91W90	TXND5	Thioredoxin domain-containing protein 5	1.48	1.32
P03995	GFAP	Glial fibrillary acidic protein	1.46	1.83
Q60631	GRB2	Growth factor receptor-bound protein 2	1.46	1.50
Q8JZX4	SPF45	Splicing factor 45	1.44	1.91
Q6P7F1	MPP4	MAGUK p55 subfamily member 4	1.37	1.89
Q61595	KTN1	Kinectin	1.33	1.79
Q8BIJ6	SYIM	Isoleucine--tRNA ligase, mitochondrial	1.31	1.43
P61082	UBC12	NEDD8-conjugating enzyme Ubc12	1.20	1.46
Q8QZY9	SF3B4	Splicing factor 3B subunit 4	1.16	1.75
Q9Z1Q9	SYVC	Valine--tRNA ligase	1.15	2.35
Q9JIF7	COPB	Coatomer subunit beta	1.14	1.75
Q9QUR6	PPCE	Prolyl endopeptidase	1.07	1.59
Q61543	GSLG1	Golgi apparatus protein 1	1.00	1.37
P60840	ENSA	Alpha-endosulfine	1.00	1.52
Q8BGT7	SPF30	Survival of motor neuron-related-splicing factor 30	0.96	1.45
O35465	FKBP8	Peptidyl-prolyl cis-trans isomerase FKBP8	0.91	1.43

Q9Z1P6	NDUA7	NADH dehydrogenase [ubiquinone] 1 alpha subcomplex subunit 7	0.90	1.31
O54983	CRYM	Ketimine reductase mu-crystallin	0.79	1.41
Q64511	TOP2B	DNA topoisomerase 2-beta	0.78	1.39
Q9JMD0	ZN207	BUB3-interacting and GLEBS motif-containing protein ZNF207	0.78	1.49
P09602	HMGN2	Non-histone chromosomal protein HMG-17	0.77	1.34
Q9EPK7	XPO7	Exportin-7	0.73	1.44
O70456	1433S	14-3-3 protein sigma	0.72	1.31
Q9QYF1	RDH11	Retinol dehydrogenase 11	0.69	1.33
Q3THW5	H2AV	Histone H2A.V	0.69	2.51
Q9CPQ3	TOM22	Mitochondrial import receptor subunit TOM22 homolog	0.67	1.33
Q61166	MARE1	Microtubule-associated protein RP/EB family member 1	0.61	2.58
Q920B9	SP16H	FACT complex subunit SPT16	0.61	2.10
**Retina_Downregulated Proteins**				
**Uniprot accession number**	**Gene ID**	**Protein Names**	**log2FC**	**-log10(p)**
O88441	MTX2	Metaxin-2	-2.99	1.34
Q9QZE7	TSNAX	Translin-associated protein X	-2.08	1.43
P62631	EF1A2	Elongation factor 1-alpha 2	-1.85	2.34
P46097	SYT2	Synaptotagmin-2	-1.84	1.75
Q923D4	SF3B5	Splicing factor 3B subunit 5	-1.79	1.40
Q99JB2	STML2	Stomatin-like protein 2, mitochondrial	-1.74	1.42
Q9WUR9	KAD4	Adenylate kinase 4, mitochondrial	-1.57	1.61
Q8K3H0	DP13A	DCC-interacting protein 13-alpha	-1.56	1.44
Q9DCB1	HMGN3	High mobility group nucleosome-binding domain-containing protein 3	-1.49	1.45
Q9DB73	NB5R1	NADH-cytochrome b5 reductase 1	-1.44	1.52
P85094	ISC2A	Isochorismatase domain-containing protein 2A	-1.26	1.91
P56380	AP4A	Bis(5-nucleosyl)-tetraphosphatase [asymmetrical]	-1.22	2.06
P55088	AQP4	Aquaporin-4	-1.20	1.35

Q8C8T8	TSR2	Pre-rRNA-processing protein TSR2 homolog	-1.17	1.57
P50153	GBG4	Guanine nucleotide-binding protein G(I)/G(S)/G(O) subunit gamma-4	-1.12	1.81
P17156	HSP72	Heat shock-related 70 kDa protein 2	-1.12	1.52
Q91WK2	EIF3H	Eukaryotic translation initiation factor 3 subunit H	-0.95	1.46
Q8BWF0	SSDH	Succinate-semialdehyde dehydrogenase, mitochondrial	-0.95	3.55
Q61503	5NTD	5-nucleotidase	-0.91	1.32
Q9D8B3	CHM4B	Charged multivesicular body protein 4b	-0.84	1.45
Q64520	KGUA	Guanylate kinase	-0.82	2.33
Q9R0Y5	KAD1	Adenylate kinase isoenzyme 1	-0.82	2.31
Q9CWS0	DDAH1	N(G),N(G)-dimethylarginine dimethylaminohydrolase 1	-0.81	1.84
Q9D3A9	TTYH1	Protein tweety homolog 1	-0.77	1.62
Q9D0D5	T2EA	General transcription factor IIE subunit 1	-0.76	1.43
O55057	PDE6D	Retinal rod rhodopsin-sensitive cGMP 3,5-cyclic phosphodiesterase subunit delta	-0.75	2.09
Q9QW08	PHOS	Phosducin	-0.66	1.74
Q9CR98	F136A	Protein FAM136A	-0.62	1.32
Q9ERD7	TBB3	Tubulin beta-3 chain	-0.60	1.86
P56480	ATPB	ATP synthase subunit beta, mitochondrial	-0.60	1.53
Q4VAE3	TMM65	Transmembrane protein 65	-0.59	1.59

Table S3. Differentially regulated proteins in optic nerve 2 weeks post Cre injection.

**Optic nerve_ Upregulated Proteins**				
**Uniprot accession number**	**Gene ID**	**Protein Names**	**log2FC**	**-log10(p)**
Q60597	ODO1	2-oxoglutarate dehydrogenase complex component E1	4.18	4.46
Q9CXW3	CYBP	Calcyclin-binding protein	3.58	2.76
Q9CR98	F136A	Protein FAM136A	3.55	2.90

Q9QX60	DGUOK	Deoxyguanosine kinase, mitochondrial	2.99	1.08
Q9Z1X2	PTSS2	Phosphatidylserine synthase 2	2.84	1.07
Q9D0I9	SYRC	Arginine--tRNA ligase, cytoplasmic	2.65	2.22
Q9JKD3	SCAM5	Secretory carrier-associated membrane protein 5	2.63	1.63
Q9CXJ4	MITOS	Mitochondrial potassium channel ATP-binding subunit	2.58	1.04
Q6PDM2	SRSF1	Serine/arginine-rich splicing factor 1	2.57	1.36
Q9CQZ6	NDUB3	NADH dehydrogenase [ubiquinone] 1 beta subcomplex subunit 3	2.53	1.59
Q9Z2W0	DNPEP	Aspartyl aminopeptidase	2.44	1.26
Q9Z1X4	ILF3	Interleukin enhancer-binding factor 3	2.07	1.44
Q9CW03	SMC3	Structural maintenance of chromosomes protein 3	2.01	1.50
Q8R0Y6	AL1L1	Cytosolic 10-formyltetrahydrofolate dehydrogenase	1.89	1.08
Q9JME5	AP3B2	AP-3 complex subunit beta-2	1.89	1.27
Q9CZ42	NNRD	ATP-dependent (S)-NAD(P)H-hydrate dehydratase	1.86	1.04
Q9DAW9	CNN3	Calponin-3	1.76	1.69
Q8CAQ8	MIC60	MICOS complex subunit Mic60	1.71	1.78
Q8VHX6	FLNC	Filamin-C	1.63	1.88
O35127	C10	Protein C10	1.59	1.64
Q80Z24	NEGR1	Neuronal growth regulator 1	1.54	1.55
P08122	CO4A2	Collagen alpha-2(IV) chain	1.50	1.93
P22599	A1AT2	Alpha-1-antitrypsin 1-2	1.49	1.85
P24668	MPRD	Cation-dependent mannose-6-phosphate receptor	1.49	1.72
Q9D1K2	VATF	V-type proton ATPase subunit F	1.42	1.21
P23336	GGTA1	N-acetyllactosaminide alpha-1,3-galactosyltransferase	1.37	1.33
Q9Z275	RLBP1	Retinaldehyde-binding protein 1	1.34	1.23
Q9D1A2	CNDP2	Cytosolic non-specific dipeptidase	1.29	2.75
Q8BFZ9	ERLN2	Erlin-2	1.26	1.05
P02802	MT1	Metallothionein-1	1.17	1.55
Q8BTM8	FLNA	Filamin-A	1.16	1.96
P26231	CTNA1	Catenin alpha-1	1.11	1.71
P16110	LEG3	Galectin-3	1.10	2.35
Q60668	HNRPD	Heterogeneous nuclear ribonucleoprotein D0	1.05	1.54

P19324	SERPH	Serpin H1	1.03	2.41
P15532	NDKA	Nucleoside diphosphate kinase A	1.02	2.03
P70202	LXN	Latexin	0.88	1.35
Q62426	CYTB	Cystatin-B	0.82	1.35
P10852	4F2	4F2 cell-surface antigen heavy chain	0.82	1.44
Q9QYB5	ADDG	Gamma-adducin	0.76	1.01
P35278	RAB5C	Ras-related protein Rab-5C	0.64	1.03
**Optic nerve_ Downregulated Proteins**				
**Uniprot accession number**	**Gene ID**	**Protein Names**	**log2FC**	**-log10(p)**
Q9ESM3	HPLN2	Hyaluronan and proteoglycan link protein 2	-1.02	4.91
Q99JP7	GGT7	Glutathione hydrolase 7	-0.94	1.42
P68368	TBA4A	Tubulin alpha-4A chain	-0.82	1.41
P09671	SODM	Superoxide dismutase [Mn], mitochondrial	-0.74	1.42
P17918	PCNA	Proliferating cell nuclear antigen	-0.72	1.40
Q62059	CSPG2	Versican core protein	-0.72	3.04
P99024	TBB5	Tubulin beta-5 chain	-0.63	2.18
P63038	CH60	60 kDa heat shock protein, mitochondrial	-0.60	1.92

Supplementary Figures

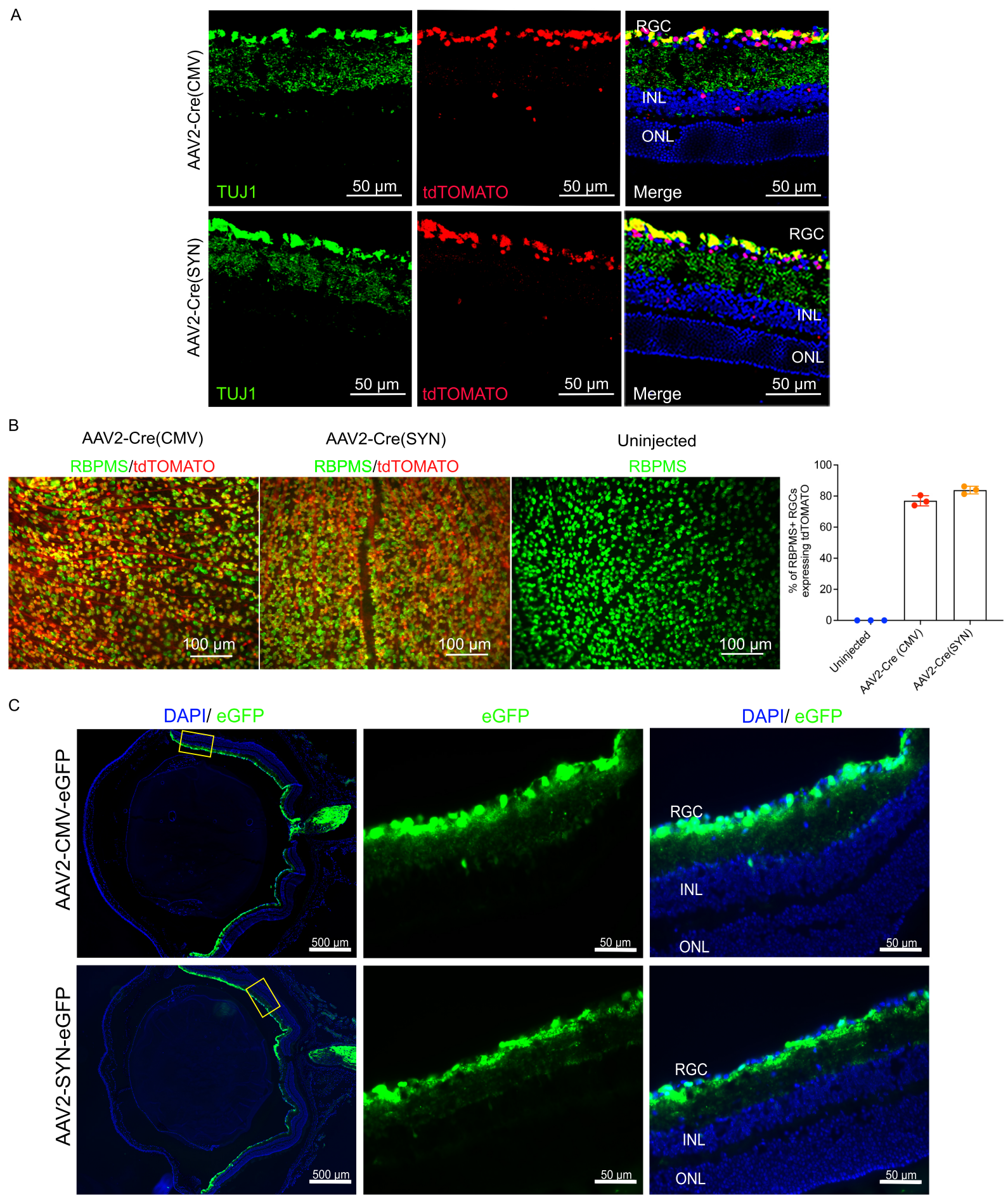

Fig S1. RGC-enriched tropism of AAV2 vectors*.* (A) Representative fluorescent microscopic images of RGC-specific expression of tdTomato (red) after intravitreal injection of AAV2-Cre under the control of CMV and SYN promoter. The sections were stained with the neuronal marker TUJ1 (green) to confirm tdTomato expression in RGCs. n=3 eyes per group; Scale bar 50 µm. (B) Ai14 (Rosa26-tdTomato) mice received intravitreal AAV2-Cre (CMV) or AAV2-Cre (SYN) and were analyzed 5 weeks post-injection. Flat-mount retinas were stained for RBPMS (green). Representative images and quantification show Cre recombination in 77.0% of $RBPMS^+$ cells for CMV and 84% for SYN, indicating robust RGC transduction by both promoters. n = 3 eyes per group; scale bar, 100 µm. (C) Cross-sections from C57BL/6 eyes injected with AAV2-eGFP (CMV) or AAV2-eGFP (SYN), processed 5 weeks post-injection. The region within the yellow rectangle is shown at higher magnification in adjacent panels. eGFP expression is predominantly restricted to the RGC layer in both conditions, however, few cells within the inner nuclear layer expressed eGFP in AAV2-Cre(CMV) injected eyes. n = 3 eyes per group; scale bars, 500 µm (low-mag) and 50 µm (high-mag).

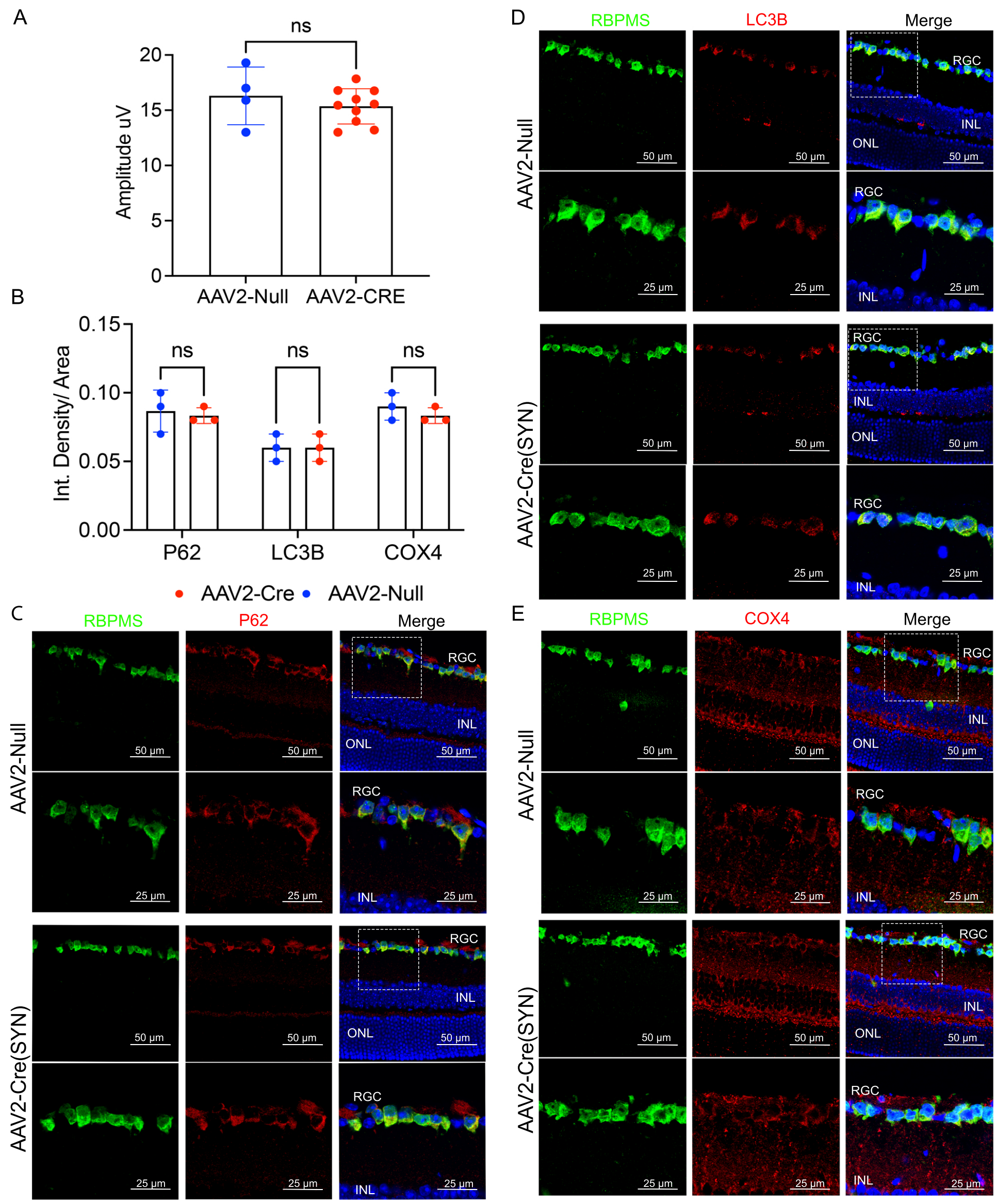

Fig S2. Cre expression does not affect RGC function or autophagy markers at 10 weeks post-injection. (A) PERG amplitudes in C57BL/6 eyes 10 weeks post-intravitreal injection with AAV2-null or AAV2-Cre show no difference, indicating no Cre toxicity to RGC function. (B) Quantified mean fluorescence intensity for p62, LC3B, and COX4 in the ganglion cell layer is unchanged between groups. (C-E) Representative confocal images of retinal sections stained for p62 (C), LC3B (D), and COX4 (E) demonstrate comparable expression in RGC layer. The area outlined by the white dashed box is shown at higher magnification beneath each panel. The enlarged view highlights RGCs in the ganglion cell layer. n = 3 eyes per group. Statistical test: Unpaired t-test (A), Two way-ANOVA (B) with multiple comparisons. *ns- nonsignificant*. Scale bars, 50 µm; insets, 25 µm.

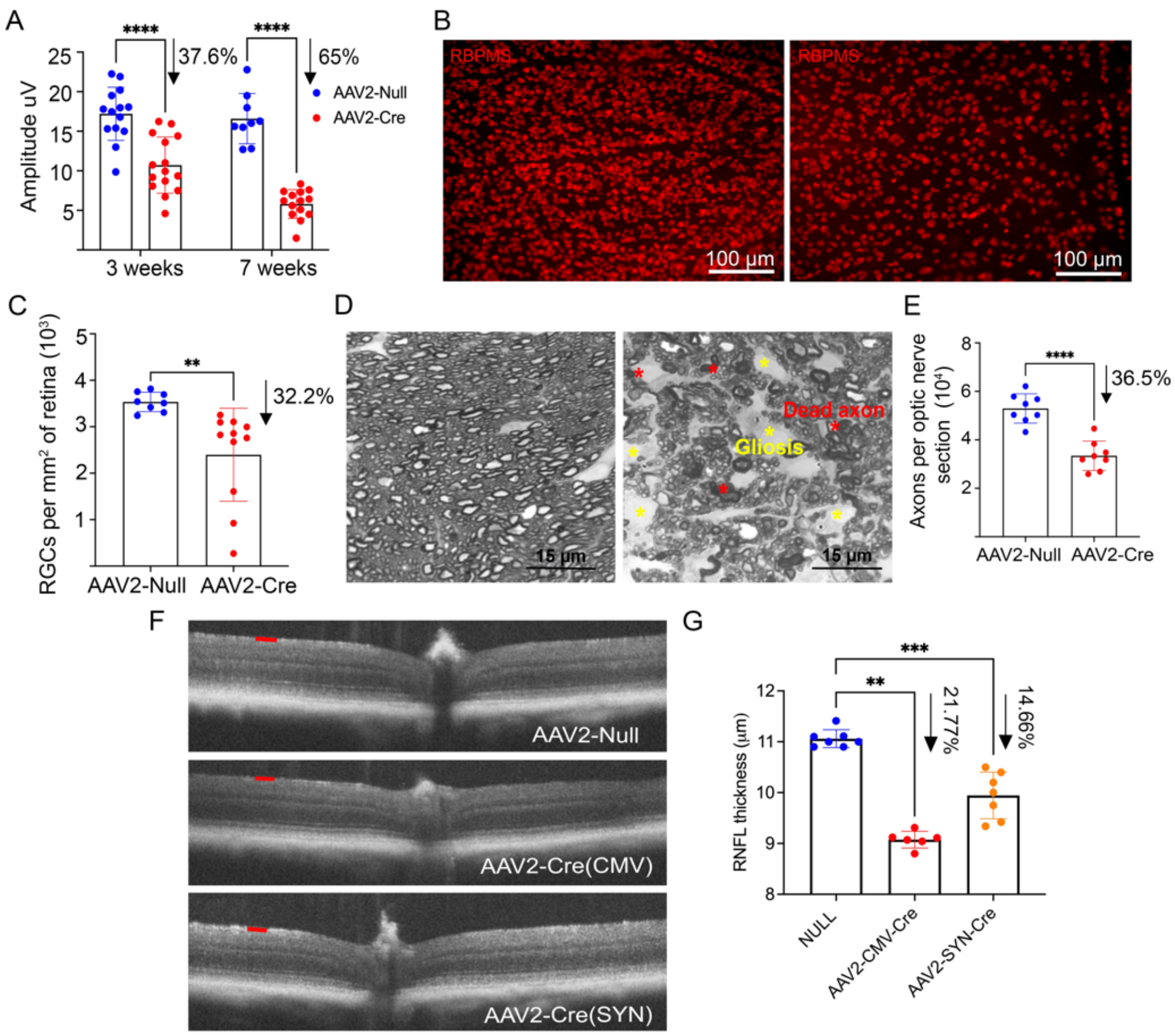

Fig S3. CMV-driven *Atg7* deletion produces early RGC dysfunction and structural loss, and RNFL thinning is observed with both CMV and SYN promoters. (A) PERG P1 amplitudes at 3 and 7 weeks post-injection in AAV2-Null versus AAV2-Cre(CMV) eyes, demonstrate an early functional reduction in the CMV cohort. (B) Representative RBPMS-stained retinal flat mounts at 7 weeks from Cre(CMV) and Null eyes. (C) Quantification of total RGC counts from the flat mounts, show a significant reduction in Cre(CMV) eyes relative to Null. (D) Representative PPD-stained optic-nerve cross-sections at 7 weeks from Cre(CMV) and Null eyes, illustrating axonal degeneration with myelin disruption and gliosis. (E) Quantification of optic nerve axon counts show a significant decrease in Cre(CMV) eyes. (F) Representative OCT B-scans from AAV2-Null, AAV2-Cre(CMV), and AAV2-Cre(SYN) eyes, with the RNFL indicated by red bars. (G) Quantification of RNFL thickness across the groups reveal significant thinning in both Cre cohorts. PERG Null n=23, Cre n=29; RBPMS Null n=8, Cre n=11; PPD n= 8 per group; OCT Null n=7, Cre(CMV) n=6, Cre(SYN) n=7.Statistical tests: two-way ANOVA with multiple comparisons for PERG (A); unpaired t-tests for RGC and axon counts (C, E); one-way ANOVA with multiple comparisons for RNFL thickness (G). Significance: ** $p \leq 0.01$; *** $p \leq 0.001$; **** $p \leq 0.0001$. Scale bars: 100 μm (B); 15 μm in (D).

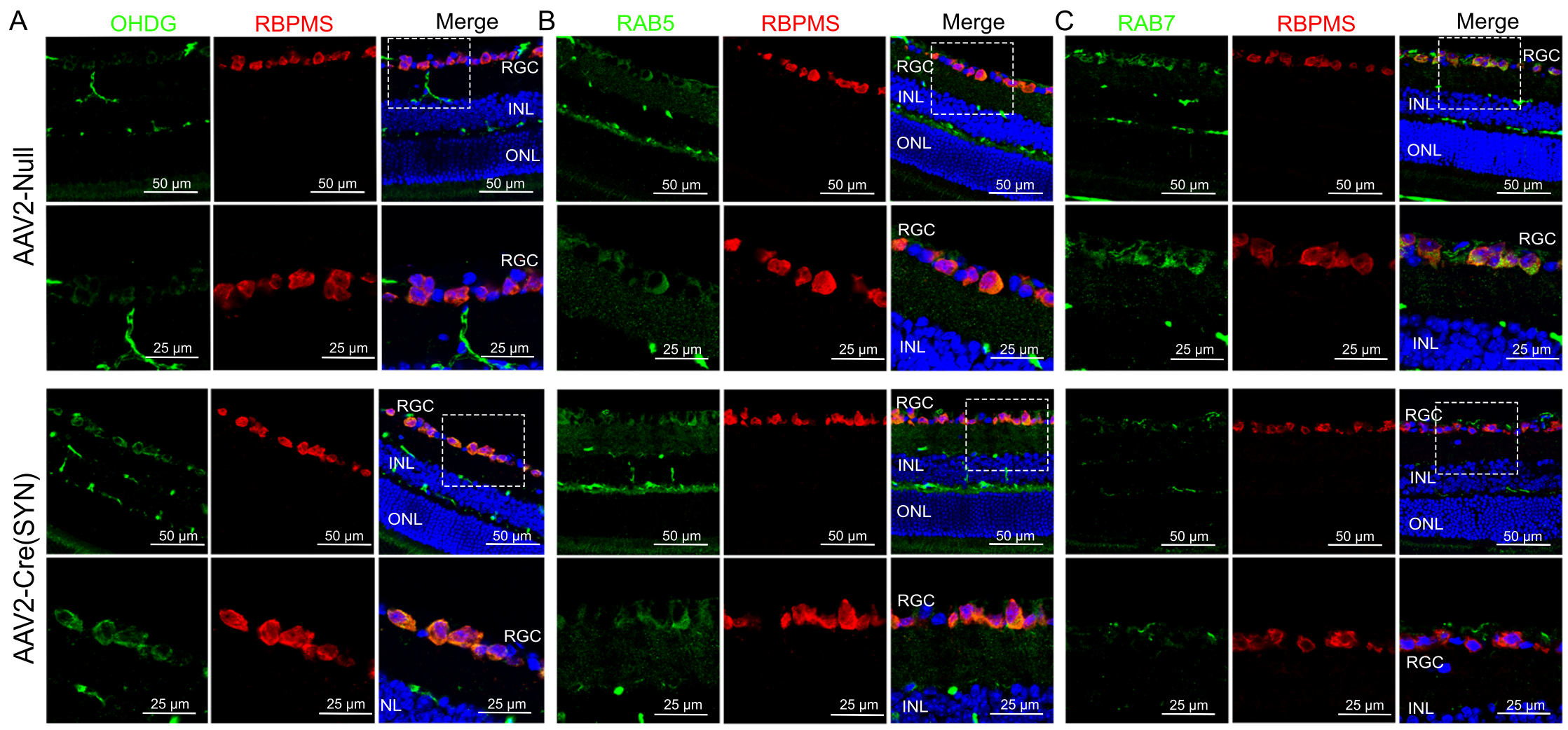

Fig S4. *Atg7* deletion increases oxidative DNA damage and lowers Rab7 in RGCs due to failed autophagosome formation*.* Representative confocal images of retinal sections from *Atg7*$^{f/f}$ eyes injected with AAV2-null or AAV2-Cre, stained for OHDG, Rab5 or Rab7 (green) with RBPMS (red) and DAPI (blue) 10-weeks post injection. (A) 8-OHdG shows increased oxidative DNA damage in Cre-injected RGCs (B) Rab5 expression in the ganglion cell layer is unchanged between groups. (C) Rab7 signal is markedly reduced in Cre-injected eyes. The area outlined by the white dashed box is shown at higher magnification beneath each panel. The enlarged view highlights RGCs in the ganglion cell layer. n = 4 eyes per group. Scale bars, 50 µm; insets, 25 µm.

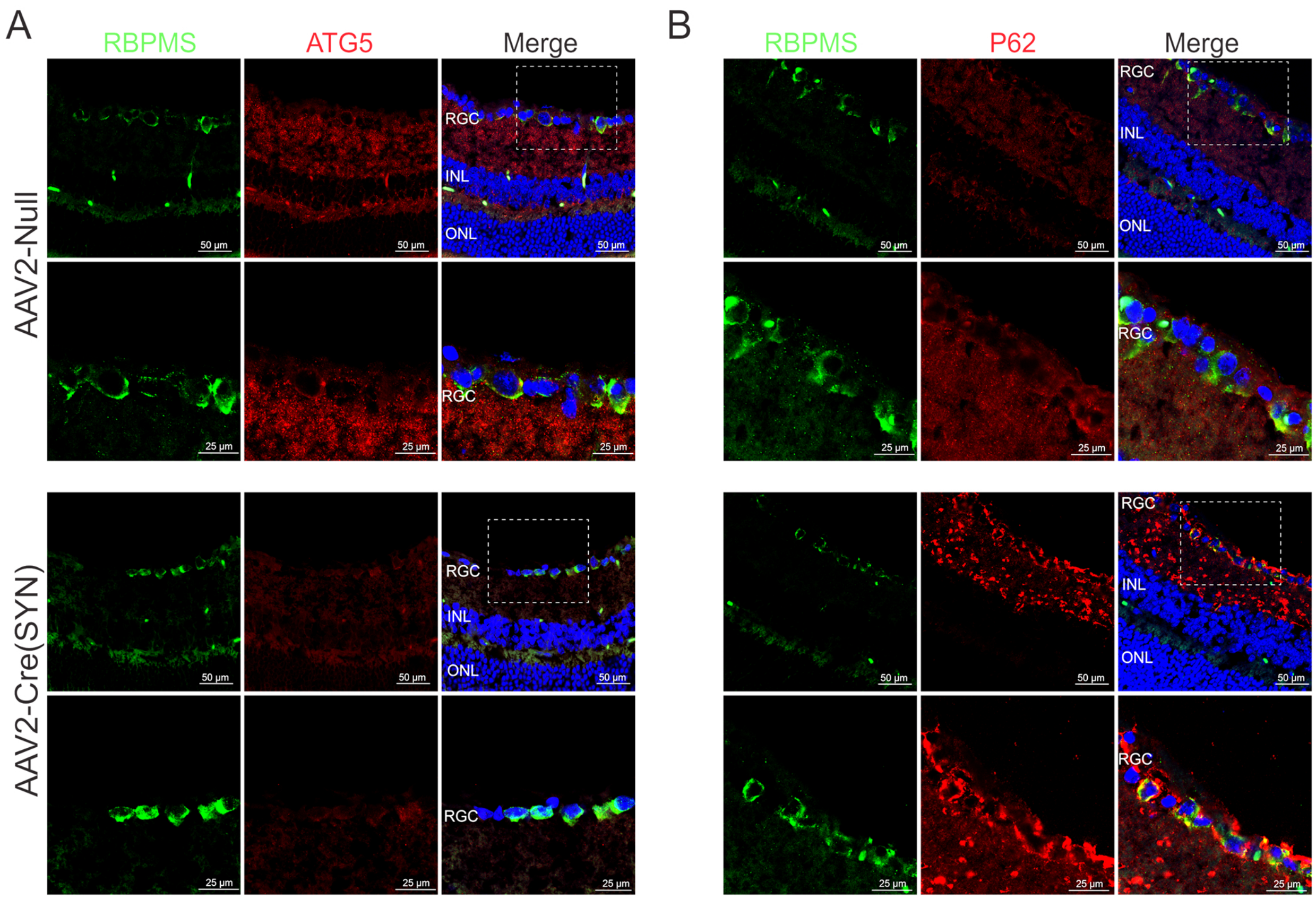

Fig. S5. Protein levels of autophagy markers in *Atg5* deficient RGCs. Representative confocal images of *Atg5*$^{f/f}$ retinal sections stained for ATG5 (A) and p62 (B) (red) together with the RGC marker, RBPMS (green). In *Atg5*-deficient (AAV2-Cre(SYN)) retinas, ATG5 immunoreactivity is reduced in RBPMS^{+} cells (A), while p62 accumulates in RBPMS^{+} cells (B), consistent with impaired autophagic clearance. The region outlined by the dashed box is shown at higher magnification below each panel. n = 3 eyes per group; Scale bar 50µm, insets 25µm.

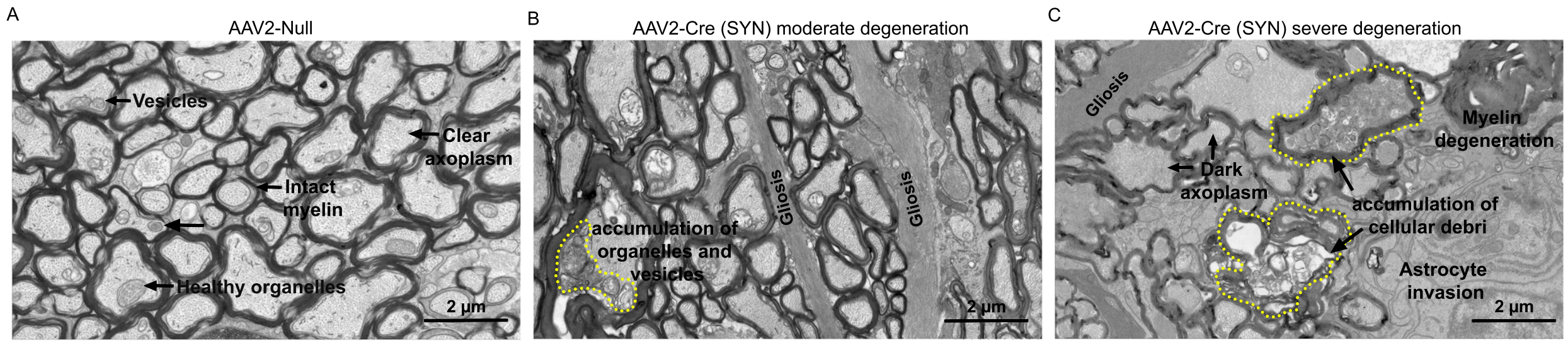

Fig. S6. Deficient autophagy leads to axonal degeneration and accumulation of damaged organelles and vesicles in optic nerve axons. To understand the ultrastructural changes in the optic nerve axons after the loss of autophagy, *Atg7*$^{f/f}$ mice were injected with AAV2-null and AAV2-Cre, and the optic nerves were processed after 7 weeks of injection for TEM imaging. Representative TEM images of optic nerve sections following AAV2-Null (A) and AAV2-Cre injection (B and C) show axonal loss and degeneration, gliosis, unhealthy and abnormal organelles, and vesicles accumulation in AAV2-Cre-injected eyes. n= 8 optic nerves per group; Scale bar 2 μm.

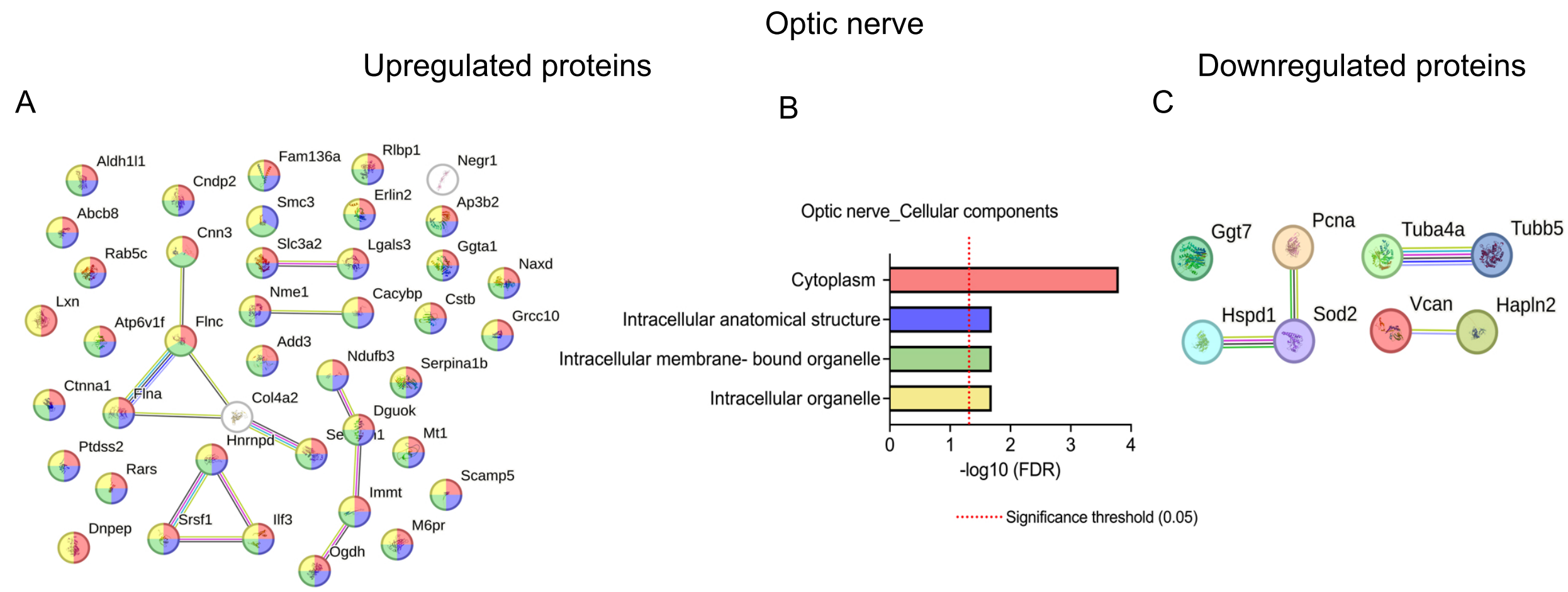

Fig. S7. Proteomic analysis showing pathways altered due to defective autophagy before neurodegeneration. To uncover molecular mechanisms potentially driving RGC degeneration, we performed proteomic analysis of the optic nerve in *Atg7*$^{f/f}$ mice two weeks after AAV2-Cre injection. This early-stage assessment, conducted before the onset of overt neurodegenerative phenotypes, aimed to identify differentially regulated proteins and associated pathways following autophagy deletion. (A) Functional network map of all proteins upregulated in the optic nerve after 2 weeks of AAV2-Cre injections. (B) Pathway analysis of proteins in (A) reveals significantly enriched cellular components in the optic nerve. (C) Functional network map of all downregulated proteins after 2 weeks of AAV2-Cre injections. n= 6 eyes per group; *FDR- False discovery rate.*
